# SRBench++ : principled benchmarking of symbolic regression with domain-expert interpretation

**DOI:** 10.1109/tevc.2024.3423681

**Published:** 2024-07-04

**Authors:** F. O. de Franca, M. Virgolin, M. Kommenda, M. S. Majumder, M. Cranmer, G. Espada, L. Ingelse, A. Fonseca, M. Landajuela, B. Petersen, R. Glatt, N. Mundhenk, C. S. Lee, J. D. Hochhalter, D. L. Randall, P. Kamienny, H. Zhang, G. Dick, A. Simon, B. Burlacu, Jaan Kasak, Meera Machado, Casper Wilstrup, W. G. La Cava

**Affiliations:** * Center for Mathematics, Computation and Cognition (CMCC), Heuristics, Analysis and Learning Laboratory (HAL), Federal University of ABC, Santo Andre, Brazil.; † Evolutionary Intelligence group, Centrum Wiskunde & Informatica, Science Park 123, Amsterdam, Netherlands.; ‡ Computational Health Informatics Program, Boston Children’s Hospital, Harvard Medical School, Boston, USA.; ¶ Heuristic and Evolutionary Algorithms Laboratory (HEAL), University of Applied Sciences Upper Austria, Hagenberg, Austria.; || Center for Computational Astrophysics, Flatiron Institute and Department of Astrophysical Sciences of Princeton University, USA.; †† LASIGE, Faculdade de Ciências, Universidade de Lisboa, Lisboa, Portugal.; ‡‡ Computational Engineering Division, Lawrence Livermore National Laboratory, Livermore, USA.; x University of Utah, Department of Mechanical Engineering, Utah, USA.; xi Meta, FAIR, France.; xii Victoria University of Wellington, School of Engineering and Computer Science, New Zealand.; xiii University of otago, Department of Information Science, New Zealand.; xiv Institut für Angewandte Physik, Universität Tübingen; Max Planck Institute for Intelligent Systems, Tübingen, Germany; xv Abzu AI, Orient Plads 1, Nordhavn 2150, Denmark

**Keywords:** Symbolic Regression, Competition, Interpretable Machine Learning

## Abstract

Symbolic regression searches for analytic expressions that accurately describe studied phenomena. The main promise of this approach is that it may return an interpretable model that can be insightful to users, while maintaining high accuracy. The current standard for benchmarking these algorithms is SRBench, which evaluates methods on hundreds of datasets that are a mix of real-world and simulated processes spanning multiple domains. At present, the ability of SRBench to evaluate interpretability is limited to measuring the size of expressions on real-world data, and the exactness of model forms on synthetic data. In practice, model size is only one of many factors used by subject experts to determine how interpretable a model truly is. Furthermore, SRBench does not characterize algorithm performance on specific, challenging sub-tasks of regression such as feature selection and evasion of local minima. In this work, we propose and evaluate an approach to benchmarking SR algorithms that addresses these limitations of SRBench by 1) incorporating expert evaluations of interpretability on a domain-specific task, and 2) evaluating algorithms over distinct properties of data science tasks. We evaluate 12 modern symbolic regression algorithms on these benchmarks and present an in-depth analysis of the results, discuss current challenges of symbolic regression algorithms and highlight possible improvements for the benchmark itself.

## I. Introduction

The goal of **symbolic regression** (SR) [1] is to find a parameterized function f(x,θ) in the form of an analytic expression that best fits the given data. Such expressions may describe non-linear interactions with a similar accuracy to that of opaque models (e.g., deep neural networks or tree ensembles), while making it possible for humans to understand their behavior in great detail like transparent models do (e.g., linear models or decision trees). Consequently, SR has been used to uncover new phenomena from collected data, extending our knowledge of physics [2], chemistry [3], medicine [4], and other fields.

Despite its utility, the SR problem is NP-hard [5]; consequently, a number of distinct fields have proposed algorithms to tackle it, from evolutionary computation to Bayesian optimization to deep learning [6, 7, 8]. In an effort to bridge existing gaps between the communities and draw a picture of the state of the art in SR, a benchmark platform called *SRBench*^1^ was recently proposed [9]. SRBench is a resource that includes different SR methods as well as other machine learning (ML) methods, and evaluates expression accuracy, simplicity, and the algorithm’s capability of re-discovering the data-generating function on physical problems. The results put forward by SRBench showed that recent SR algorithms based on genetic programming (GP) tend to be the best performing on real-world tabular problems. Moreover, SRBench contributed to a growing body of literature demonstrating that SR approaches perform as well as or better than ML methods commonly accepted to be the state-of-the-art (SotA) for this task [10, 11, 12].

Notwithstanding the importance of such results, a number of interesting questions on SR remain unanswered to date. For example, SRBench does not evaluate how well each SR algorithm behaves with respect to distinct *properties* of real-world data, such as presence of noise, redundant features, or extrapolation behavior outside of the training distribution. Furthermore, SRBench, like many benchmarks of SR algorithms, has a limited ability to benchmark the interpretability of models, which frequently requires subjective input from model users with domain expertise.

In this paper we describe an extended benchmark, SRBench++, that complements and extends SRBench by proposing specific tasks not studied in the original benchmark, and soliciting expert feedback for evaluating model rankings. The proposed benchmark integrates with the SRBench framework and provides a separate focus from prior work. Whereas SRBench focused on the expected rank of an SR algorithm w.r.t. an arbitrary dataset, SRBench++ verifies how well each algorithm behaves when challenged with specific tasks. To that end, we considered a number of assessment criteria that go beyond expression accuracy and length: *feature selection, sensitivity to local optima, accuracy in the extrapolation regime, sensitivity to noise, rediscovery of known laws* and, importantly, *interpretability* according to an expert of the field for a real-world use, i.e., forecast of key indicators for the COVID-19 pandemic. To ensure the comparison was fair, we asked the participants to submit their algorithms through SRBench system while concealing these new benchmarks. As such, participants did not have any prior information regarding the tasks. This resulted in the evaluation of 12 distinct algorithm submissions based on GP, deep learning, and combinations thereof, developed by research teams spanning industry and academia.

In the following sections, we describe the rationale behind our design choices for SRBench++ and report an in-depth analysis of SR algorithms on this new benchmark. We leverage this analysis to identify current research challenges and opportunities for SR with respect to data science applications as well as benchmarking. In Section II we give a brief introduction to SR and the current SotA. Section III we describe the benchmark rules, tasks, evaluation and competitors. In Section IV we show and analyse the obtained results with focus on the winning entries. Finally, Section V discuss the results summarizing the insights obtained from this benchmark and we emphasize the current challenges of SR research. We conclude this paper with some final thoughts and future steps in Section VI.

## II. Background

SR is the problem of searching for a closed-form expression, i.e. that best fits the available data by composing simpler functions (often called primitives) from an user-defined set. This search is usually guided by a loss function ℒ(y,f(x,θ)) that measures the approximation error of the regression model, f(x,θ), according to the measured target, y. The main advantages of creating an analytic expression are the possibilities for manual inspection, debugging, and adaptation by the practitioner. This manual manipulation can lead to a better understanding of the model, improve the accuracy of the model, or allow specialists to incorporate expert knowledge. Apart from the manual manipulation, we can also analyse the behavior of the model using common regression tools [13, 14]. Although simple models based on analytic expressions may be overlooked in lieu of complex, opaque models, they often exhibit similar accuracy in practice [9, 15]. Specifically for SR, there is evidence that analytic expressions excel at extrapolating outside the training data region in comparison to gradient boosting and neural networks [16].

Although SR has some advantages over other regression methods, until recently, the adoption of SR in practice has been limited for a number of reasons:

- longer training time compared to traditional regression;
- a lack of implementations in easy-to-use toolboxes;
- the difficulty of customizing existing toolboxes;
- a lack of supporting tools to interpret generated models;
- a smaller and more insular research community compared to other ML fields; and
- a lack of shared dependencies / platforms between methods and toolboxes.

Attempts to address these limitations began with Orzechowski et al. [10] and led to the creation of SRBench [9]. SRBench not only revealed the benefits of using SR for prediction tasks but also created an environment that enables practitioners easy access to different SR approaches using a *scikit-learn*-like Python interface.

## III. SRBench++ : a task-driven benchmark for Symbolic Regression

The goal of SRBench++ was to assess different aspects of SR algorithms other than prediction accuracy, to understand the current challenges of SR, and also to further stimulate the growth of SRBench, which is intended to be a reference and ever-improving benchmark for tracking the SotA in the field. We structured the benchmark into three distinct stages. First, a qualifying stage was used to filter methods that did not meet a minimal level of performance, defined as matching/exceeding the accuracy of ordinal least squares on a subset of publicly available datasets. After the qualifying stage, the benchmark proceeded in two tracks: a synthetic track to assess different properties of interest measured against ground-truth solutions, and a real-world track, where an expert was asked to judge competing models of COVID-19 spread using publicly available health data. Qualifying methods were evaluated on both tracks.

For each one of the 10 individual runs, we specified a random seed and the candidate SR algorithm had a pre-specified time budget of 1 hour for datasets up to 1000 samples and 10 hours for datasets up to 10000 samples. Within this time budget the candidate could perform any pipeline they wanted (i.e., data pre-processing, hyperparameter optimization, etc.). Candidates were responsible for ensuring that the runtime of their pipeline would not exceed the budget. In order to make comparisons as fair as possible, participants were not given advance notice of what datasets would be used for the benchmark. They knew only that datasets would follow the format of the Penn Machine Learning Benchmark (PMLB) [17, 18], and that datasets from PMLB would be used in the qualifying stage.

With the objective of making the benchmark accessible and reproducible by external peers, we created a new branch in the SRBench repository^2^ with instructions on how to add a new method and how to generate the results on a local machine. We solicited entries for this benchmark using a competition held at the GECCO 2022 conference in Boston, MA. We accepted both to open- and closed-source entries, provided that the algorithm provided an API compatible with SRBench. Participants submitted their own algorithms without any knowledge about the datasets or the tasks. Entries were considered “official” once they passed a series of automated tests facilitated via Github actions.

### A. Qualification Track

In the qualification track, we used a selection of 20 PMLB datasets to verify whether the competitors were capable of finding better models than a linear model baseline. The main objective of this track was to filter entrants that did not adhere to a minimum acceptable prediction accuracy. The evaluation criteria for this track was the median $R^{2}$ score on 10 distinct runs. We ranked the results by the median across the different datasets and disqualified those approaches that were ranked lower than using plain linear regression. The chosen datasets are detailed in the supplementary material.

### B. Synthetic tracks

For the synthetic tracks, our goal was to evaluate different tasks that simulate challenges often observed in real-world data. By evaluating such challenges, we hope to gain a more granular understanding of specific tasks for which current algorithms excel or have room for improvement.

In these tracks, we used multiple evaluation criteria: $R^{2}$, simplicity, and a task-specific score for each tasks. We then computed the aggregated rank as the harmonic means of the ranks for each criterion for n different data-sets. The harmonic mean imposes that, to be highly ranked, you cannot have a low rank in any of the criteria. This avoids the situation that an SR algorithm returns a very simple model with low $R^{2}$ and still ranks high among the competitors.

The simplicity score is defined as $\text{round}\left(-{\text{log}}_{5}(s),1\right)$, where s is the number of nodes in the expression tree after being simplified by *sympy* [19], and round(x,n) rounds the value x to the n-th place. Rounding was introduced to provide some tolerance for similarly-sized expressions.

We tested 5 different tasks in this track:

1. **Rediscovery of the exact expression:** the SR model must match the exact generating function of the dataset.
2. **Selection of relevant features:** the SR model must only use relevant features, discarding any noisy feature.
3. **Deceptive shortcuts:** the SR model should be composed of noise-free, low-level features instead of noisy interactions of the original features.
4. **Extrapolation accuracy:** given a dataset with a limited range on the variables’ domains, the SR model should behave as intended outside this range.
5. **Sensitivity to noise:** given different levels of noise applied to the target value, the SR algorithm should recover an expression close to the original function.

In the following subsections we will explain each one of these tasks in further details with the data generating process and evaluation criteria.

#### 1) Rediscovery of exact expression:

In this task, the SR algorithm should return a model that is within a constant (multiplicative or additive) factor of the generating function. To assess this, we rely on the ability of *sympy* to manipulate expressions algebraically and evaluate the equivalence of two expressions. The specific property measure is:

$$\text{exact}\left(f_{\text{true}},f_{\text{pred}}\right)=\left\{\begin{array}{cc} 1 & \text{if}\;\text{isNumber}\left(f_{\text{true}}-f_{\text{pred}}\right)\\ & \text{or}\;\text{isNumber}\left(f_{\text{true}}/f_{\text{pred}}\right)\\ 0 & \text{otherwise} \end{array}\right.$$

where isNumber returns true if the expression is a constant value and false otherwise. Assuming that the results of the algebraic –, / operators will be simplified. The idea of this measure is that if $f_{\text{pred}}$ is equal to $f_{\text{true}}$ except for an additive or multiplicative constant value, it will return success (1), otherwise it fails (0). The main drawback of this measure is that it cannot measure how close the expression is from the ground-truth and it will not detect if an expression is equal to the true but with an additive and multiplicative constant.

For this task, we have tested four increasingly difficult generating functions departing from a base function $\left(f_{1}\right)$ and introducing nonlinear terms. Table I shows the corresponding generating functions.

#### 2) Selection of relevant features:

For this task, we created datasets with 20 features following the generating function:

(1) $$0.11x_{1}^{3}+0.91x_{3}x_{5}+0.68x_{7}x_{9}+0.26x_{11}^{2}x_{13}+0.16x_{15}x_{17}x_{19}.$$

As we can see from Eq. 1, the variables with an even index are not used when generating the dataset (they are random variables unrelated to the function). The main goal is that the SR model identifies and uses only the odd indexed features. For this purpose, we introduce the specific score for this task given by:

(2) $$\text{feature-select}\left(f_{\text{true}},f_{\text{pred}}\right)=\frac{\text{false-feats}-\text{false-sel-feats}}{\text{false-feats}},$$

where false-feats is the set of random valued features and false-sel-feats is the set of those noisy features used by the SR model. This measure is equivalent to the Jaccard Index between the set of false features and the set of selected false features and will return 1 if the model does not use any noisy feature and 0 if it uses all of them. There are also three difficulty levels in this task – easy, medium, hard – that are created by adding different level of noise to the target value and replacing the target with:

(3) $$y_{\text{noise}}=𝒩\left(y,\sigma_{y}\sqrt{\frac{\text{ratio}}{1-\text{ratio}}}\right)$$

where ratio is the noise level. The ratio of each difficulty setting is 0.025 for easy, 0.05 for medium, and 0.1 for hard.

#### 3) Deceptive shortcuts:

For this task, the dataset contains the original features and some meta-features that are higher-level building blocks of the generating function. However, crucially, the data regarding the meta-features contains noise. Therefore, a good but suboptimal model can be constructed by combining the meta-features instead of the original features. We defined the meta-features (without noise) as:

(4) $$f(x)=\underset{g_{1}(x)}{\underbrace{0.77x_{1}x_{2}}}+\underset{g_{2}(x)}{\underbrace{1.52x_{2}x_{3}}}+\underset{g_{3}(x)}{\underbrace{1.2x_{4}^{2}}}+\underset{g_{4}(x)}{\underbrace{0.31x_{1}x_{4}x_{5}}}+\underset{g_{5}(x)}{\underbrace{0.23x_{3}x_{4}x_{5}}}.$$

and the generating function with n meta-features is given by $f_{n}(x)=\sum_{i=1}^{n}g_{i}(x)$.

The corresponding dataset is built as a list of samples in the format $\left[x_{1},x_{2},x_{3},x_{4},x_{5},g_{1}+\epsilon ,\ldots ,g_{n}+\epsilon ,f_{n}(x)\right]$, where ϵ is an additive noise following Eq. 3 with a ratio of 0.1. Since noise is added to the data of the meta-features g, the optimal fit to $f_{n}$ can only be recovered if the meta-features are ignored, and the original features x are used. There are also 3 levels of difficulty controlled by the value of n: easy with n=3, medium with n=4, hard with n=5. The task-specific score used here is the same as the one for the feature selection task.

#### 4) Extrapolation accuracy:

The goal of this tasks is to evaluate the performance of SR models when the validation set is outside the training data domain. This is a tricky situation as by fitting the model on a limited space there can be many equally good models that behaves differently outside the training boundaries. Models are thus rewarded for choosing a minimally complex hypothesis among those describing the training data. For this task we used the generating function:

(5) $$f\left(x\right)=\text{erf}\left(0.22x\right)+0.17\;\text{sin}\;\left(5.5x\right),$$

where erf is the error function defined as

$$\text{erf}\left(z\right)=\frac{2}{\sqrt{\pi }}\int_{0}^{z}e^{-t^{2}}dt.$$

Besides not having an analytic solution to this problem, the training and validation datasets were split such as -15≤x≤15 for the training set and 15<x≤40 for the validation set.

The specific measure for this task is the evaluation of the $R^{2}$ score on the validation data. Like in the other tasks, we created three different level of difficulties by adding noise following Eq. 3 with ratios 0.05 (easy), 0.1 (medium), and 0.2 (hard).

#### 5) Sensitivity to Noise:

In this task the SR models are created using a noisy training data and evaluated on a noiseless validation data. The generating function used was:

$$f\left(x\right)=\frac{0.11x^{4}-1.4x^{3}}{0.68x^{2}+1}.$$

The different noise ratios were 0.05 (easy), 0.1 (medium), and 0.15 (hard).

### C. Real-world track

For the real-world track, the objective was to evaluate the interpretability of SR in a real-world scenario, rather than relying on measures of complexity. In real-world scenarios, the interpretability of a model can be subjective and depends on the expertise of specialists from the domain of application. To this end, we tested the ability of competitor methods to generate explainable predictive models of COVID-19 cases, hospitalizations, and deaths.

**Fig. 1.**
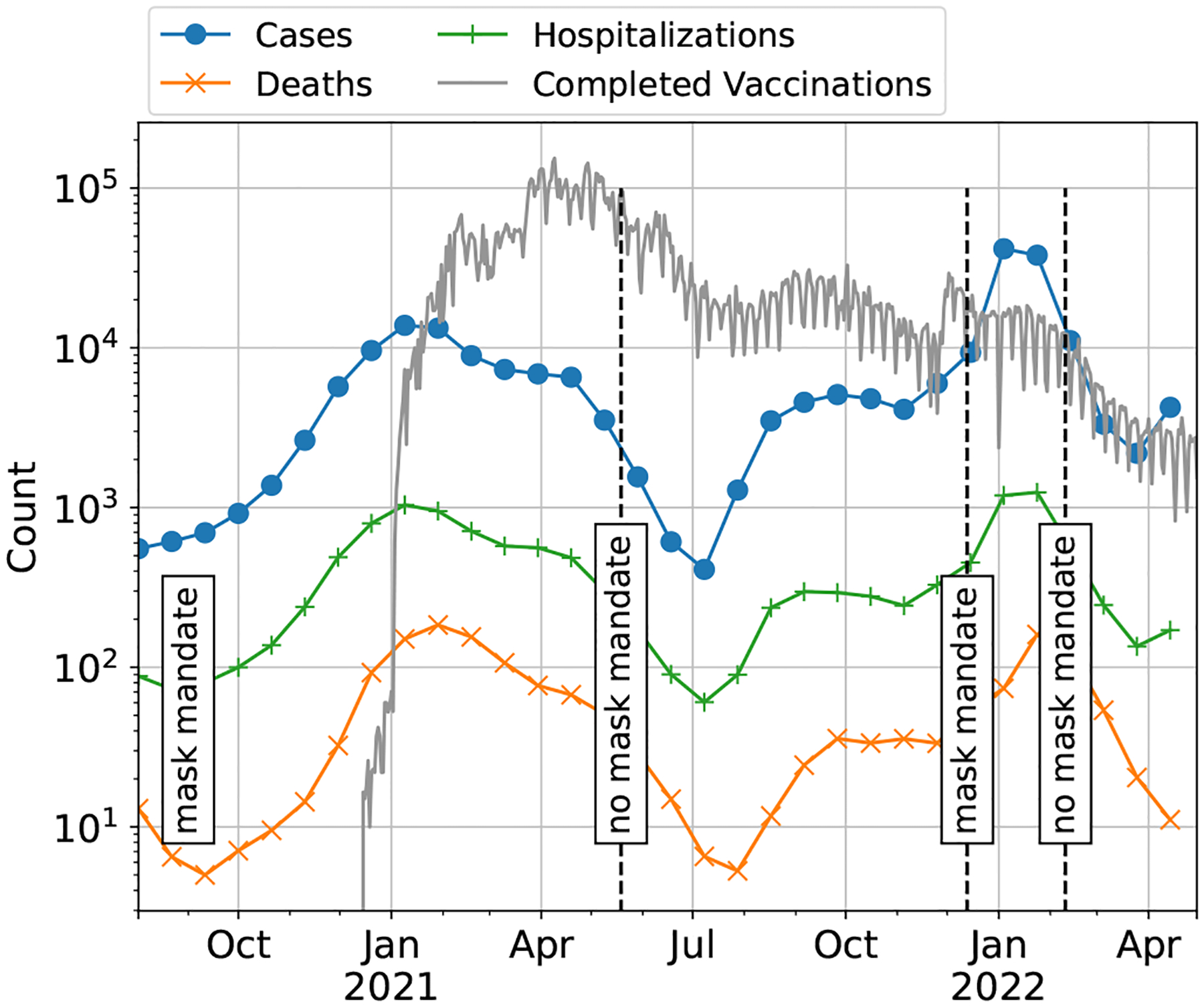
COVID-19 data in New York state from August 2020 to April 30, 2022, used for the real world track. The data consist of COVID-19 cases, deaths, hospitalizations, completed vaccinations, and policy indicators for mask mandates (timing shown above) and school closures (not pictured).

The task was constructed using time-series data for daily numbers of cases, hospitalizations, and deaths in New York state between August 2020 and April 30, 2022. We utilized an existing collation of these data from the Github repository https://github.com/reichlab/covid19-forecast-hub/. In addition, we collected data on covariates hypothesized to be associated with disease outbreak, including policy measures (mask mandates, school closures) and daily and total vaccinations. A summary of the data is given in Fig. 1. Each method was tasked with projecting future counts of cases, hospitalizations, and deaths using historical data.

Time series data were preprocessed by replacing outliers (±4σ) with the median value in a one week window. Afterward, we smoothed the measurements with an exponentially weighted moving average.

Models were developed to predict the count of cases, hospitalizations or deaths with a two week horizon using data from the prior two weeks (i.e., 14 day look-ahead models with a 14 day lag). The lag and lookahead window were chosen based on discussions of utility with the expert reviewer. At time t, the target label corresponds to $y_{t+h}$ where h is the prediction horizon. For each prediction time t, each time series x, and each day in the lag period i, we extracted three features: 1) the observed value i days prior, i.e. $x_{t-i}$; 2) the difference between the current value and the value i days prior, i.e. $x_{\Delta i}$; and 3) the cumulative sum of the values until the current timestep, $x_{\text{tot}}$.

The training and test sets were created by alternating between 5 weeks and 3 weeks chunks, respectively, so that the algorithms would have samples from the entire duration of the data collection.

All the algorithms were executed for 10 times for each dataset, resulting in 10 models per algorithm. We selected the best model w.r.t. the $R^{2}$ score on the test set as the representative model for each candidate algorithm. The best models were evaluated by an infectious disease expert (Dr. Majumder) using a trust score ranging from 1 (strong distrust) to 5 (strong trust). The candidate model’s final score was taken to be the harmonic mean between accuracy, simplicity, and this expert trust score.

### D. Algorithm entries

A total of 13 teams participated in the benchmark, including 1 withdrawal. For brevity, all methods are summarized in Table II. We report a description of each method submitted by the participants in the Supplementary Material. We note that GP_ZGD_ was a late submission, and was only evaluated in the qualification stage of the benchmark.

## IV. Results

This section presents a detailed analysis of the results for each track of the benchmark. A summary of these results can be found at the benchmark website^3^.

### A. Qualification Track

**Fig. 2.**
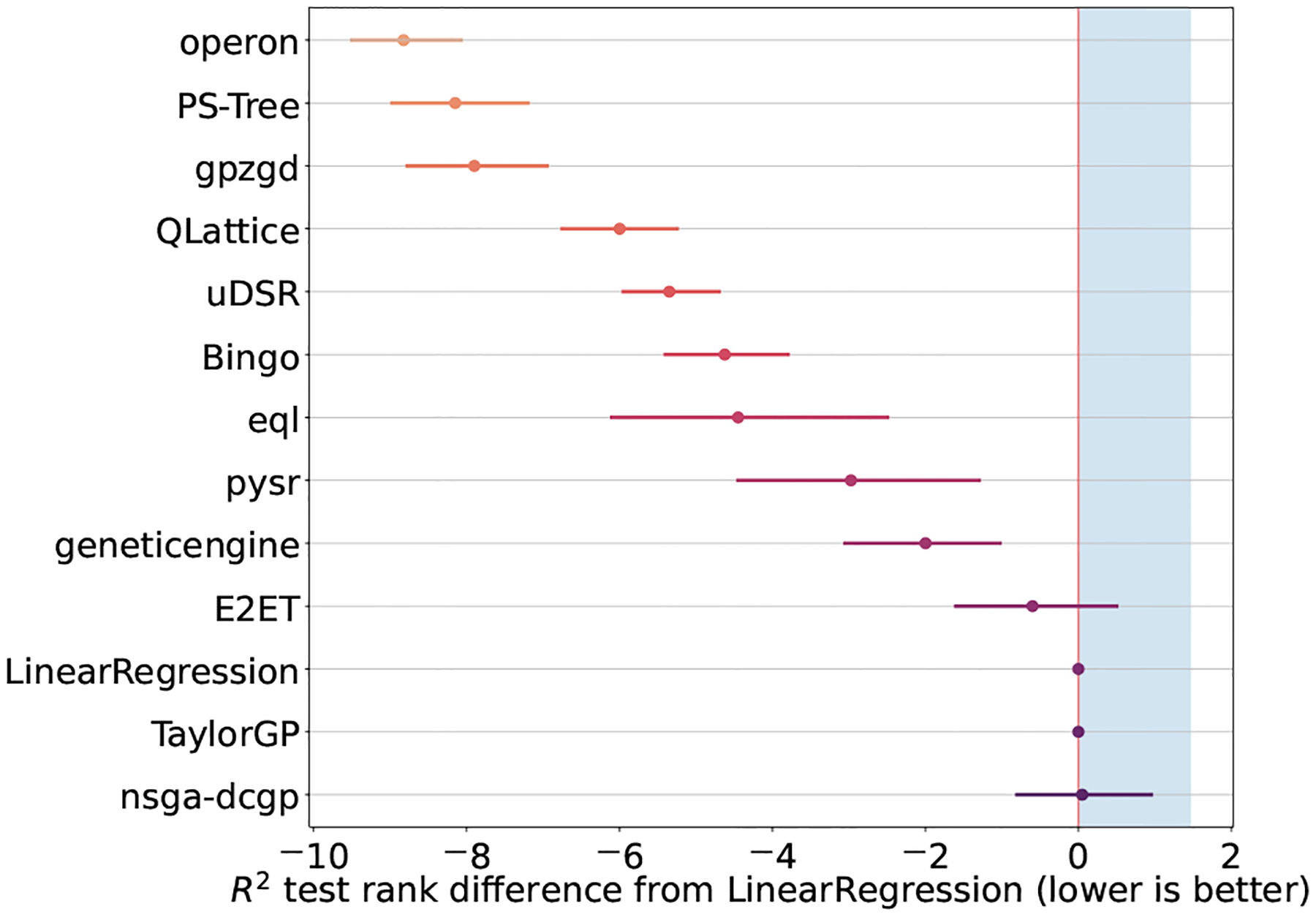
Rank of the SR methods for the qualification track calculated as the median $R^{2}$. In this plot, the lower the value the better. The shaded area depicts the disqualified competitors.

The rank of the tested SR methods for the qualification track is reported in Fig. 2. From this figure we can see that most of the competitors found better models than the linear regression. In this stage, the SR methods TaylorGP and nsgadcgp were disqualified. The former simply returned linear models while the latter returned some slightly better models and some slightly worse than linear regression. Even though E2ET was better than linear at the median score, it also found a worse than linear model in some occasions. One reason being that this algorithm can only handle datasets up to 10 variables and some of those datasets had more than that. On the other side of the spectrum, we have Operon, PS-Tree and GP_ZGD_ as the most accurate models for this selection of datasets.

### B. Synthetic Benchmark Track

**Fig. 3.**
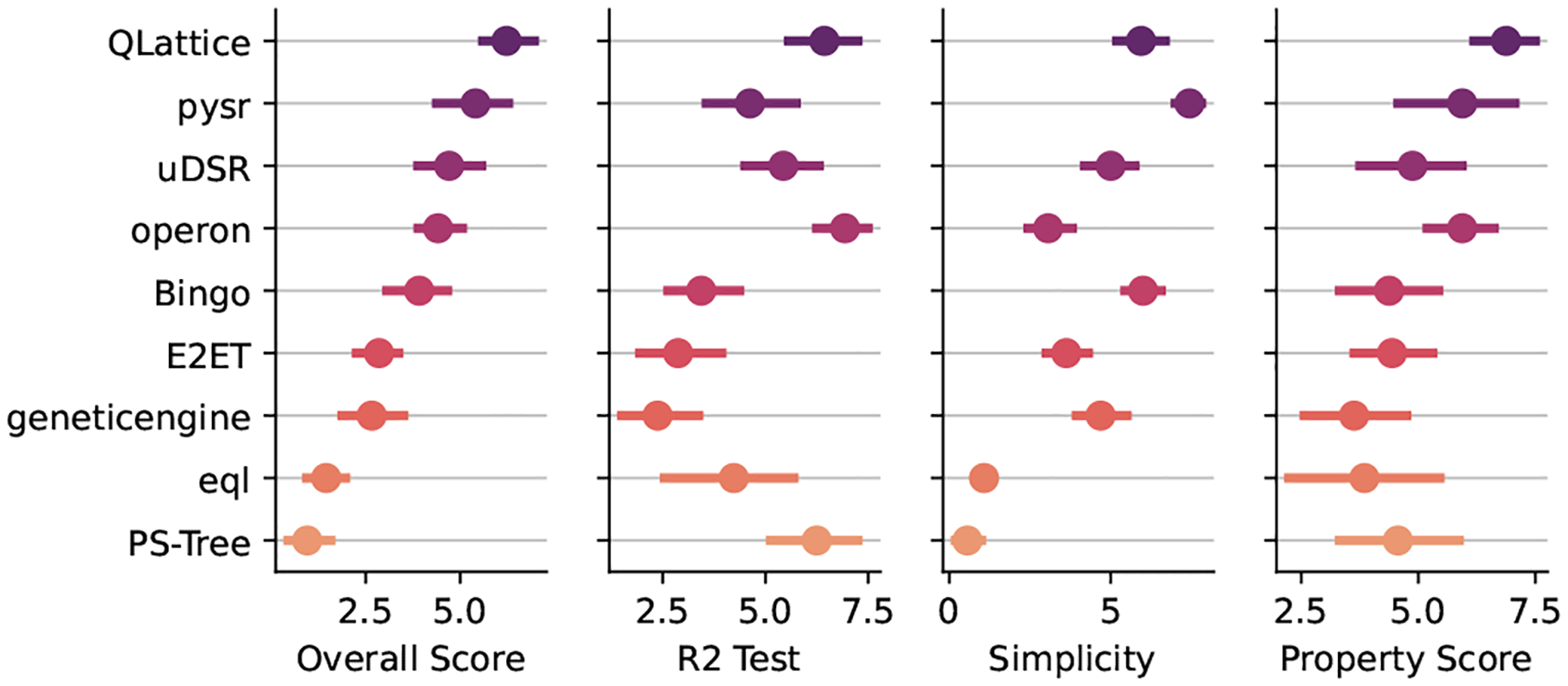
Aggregated results for all tasks in the Synthetic Benchmark Track. The leftmost plot presents the distribution of the harmonic mean of all three criteria, the medians of this plot was used to decide the winner. The next three plots shows the distribution of $R^{2}$, simplicity, and task-specific, respectivelly.

Fig. 3 shows the aggregated rank comprising every task of the synthetic benchmark track. As we can see from this figure QLattice ranked first, followed by pysr and uDSR when considering the harmonic mean of all three criteria. Notice that the higher the value of the rank, the better; the reason for this is that the harmonic mean penalizes small values more, as per our intention to penalize models that does not satisfy all criteria. We can also see from this plot that there is an overlap in the distribution among the contenders, suggesting that the results may not be statistically significant.

Regarding $R^{2}$ score, operon returned the best models followed by QLattice and PSTree. Looking at the simplicity scores, pysr, QLattice, and Bingo were the top three, and for the property specific score the best algorithms are QLattice, pysr, and Operon. QLattice is among the top-3 in every criteria, corroborating with the first place in the harmonic mean of the ranks.

We next tested for statistically significant differences in harmonic mean scores among algorithms across all the synthetic track benchmarks. Supplemental Fig. 2 shows the critical difference diagrams, using the Nemenyi test (α=0.05). When considering all tasks together, the top five Ranking algorithms did not exhibit significance differences among their results.

#### 1) Rediscovery of exact expressions:

**Fig. 4.**
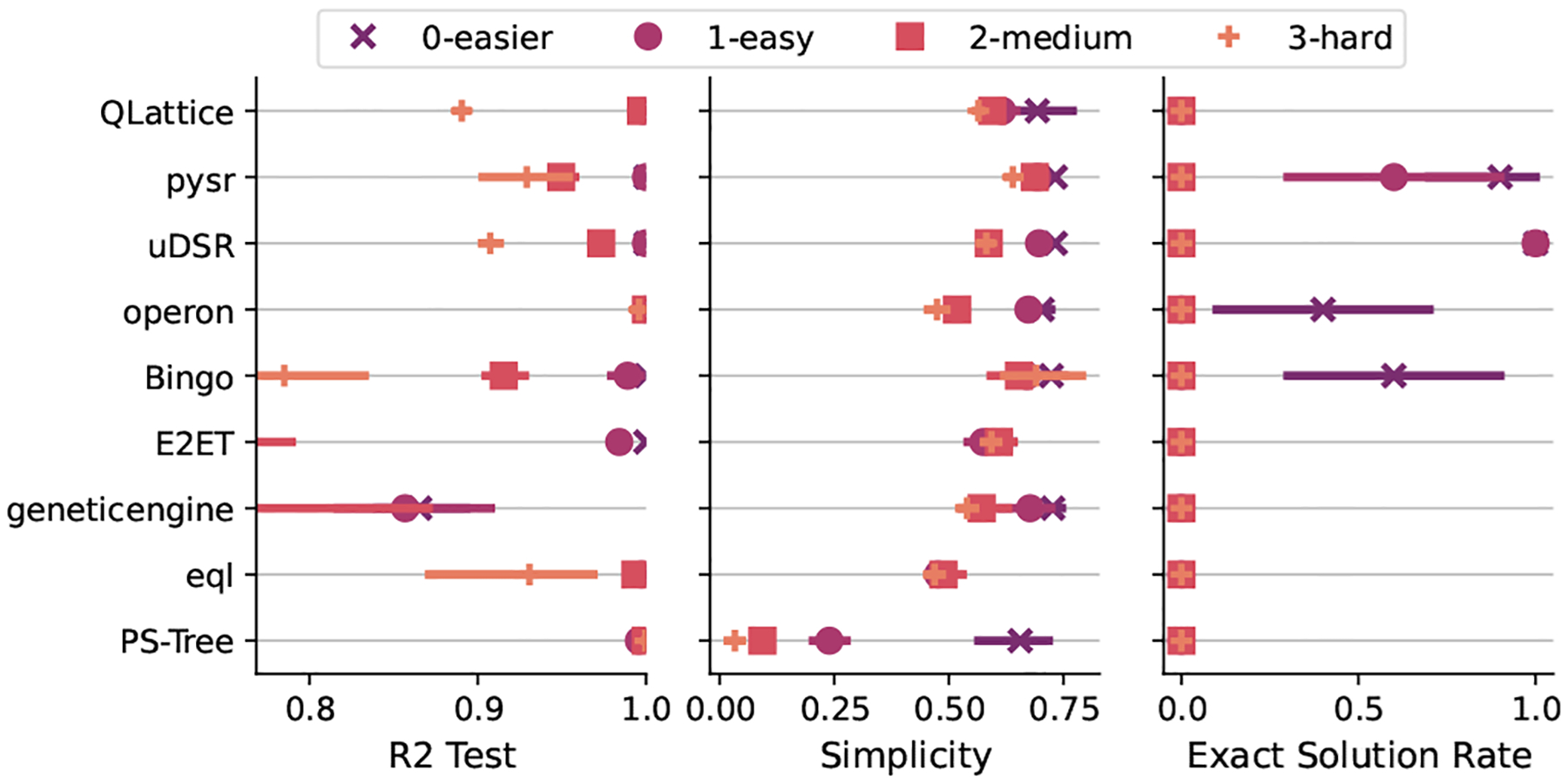
Distribution of ranks for the discovery of exact expressions. Higher rank values (to the right) are better.

In Fig. 4 we can see the results for the rediscovery of exact expression task grouped by the different levels of difficulties. The first observation is that most of the algorithms are capable of finding accurate models (with $R^{2}$ higher than 0.9) on every level of difficulty, the only exceptions being Bingo, geneticengine, E2ET on the higher difficulty levels. They are also capable of maintaining a similar level of simplicity in comparison to each other, except for PS-Tree that created more complex models.

Regarding the recovery of the expression, we can see that this was only achieved for problems in the easier and easy difficulty levels. Further, only pysr, uDSR, operon, and Bingo were capable of finding exact solutions in these levels. It should be highlighted that uDSR found a perfect match in every run for those two levels.

By selecting the model with the highest harmonic mean rank between the three criteria, we found models very close to the ground-truth obtained by Bingo and pySR. For the easier level, Bingo found the following expression:

$$f(x)=0.4\times \left(x_{1}+6.25\right)\times \left(x_{2}-3.75\right)+10.37$$

that matches the generating function exactly after some algebraic manipulation.

The other two difficulty levels were particularly hard for the SR algorithms. One possible reason is that SR usually have difficulties handling rational functions due to using protected division or replacing the division operator by the analytic quotient to avoid partiality. Using the same criteria as above, the best model returned by operon for the medium difficulty level resembled the true expression:

(6) $$f(x)=\frac{-1.66x_{1}+\left(-0.02x_{1}-0.15\right)\left(-16.15x_{2}-6.46\right)}{\left(0.2x_{1}^{2}+1\right){\left(\frac{0.19x_{2}^{2}}{0.2x_{1}^{2}+1}+1\right)}^{0.5}{\left(\frac{0.21x_{2}^{2}}{0.2x_{1}^{2}+1}+1\right)}^{0.5}}$$

If we change $0.19x_{2},0.21x_{2}$ both to $0.2x_{2}$, we get an expression that, after some algebraic manipulation, becomes the true expression (Table I). This reveals some problems related to the internal optimization of the numerical parameters that can hinder the search for the correct expression: i) the expression may be ill-conditioned [31]; ii) it may not have reached the local optimum (computational budget and accuracy trade-off); iii) it may have converged to a bad local optimum; iv) it can bias the search to overparametrized expressions [32]. This limits the application of an algebraic simplification to alleviate this problem, as seem in the previous example.

#### 2) Selection of relevant features:

**Fig. 5.**
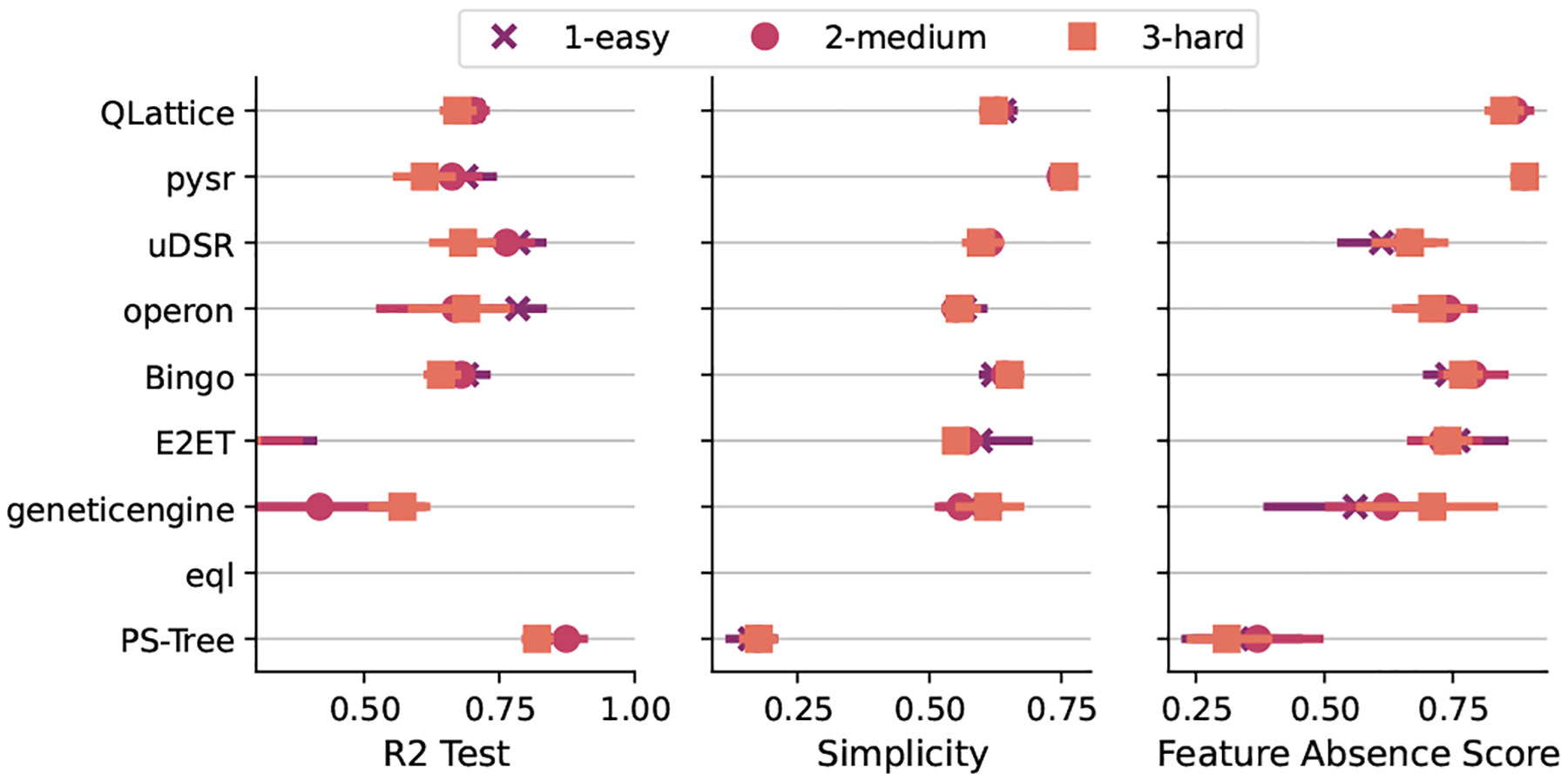
Distribution of ranks for the feature selection task. Higher values are better.

Fig. 5 shows the distribution of ranks for the task of using only the relevant features in the model. Analysing the common criteria for all tasks, we can see that PS-Tree stands out as the most accurate model and the most complex one at the same time, for every difficulty level. Incidentally, it is also the algorithm with the smallest scores for the feature absence score. Being a piecewise approach, it is likely that it created additional sub-expressions to account for the additive noise, generating a more complex expression and making use of almost every feature.

The algorithm pysr obtained the best harmonic mean for all three levels of difficulty with this model:

$$f\left(x\right)=7.1x_{1}+x_{2}x_{3}+0.27x_{6}^{2}x_{7},$$

which is very different from the true generating function.

As we can see from this equation, the pysr model uses two irrelevant features and misses some of the true features. That being said, we should stress that none of the algorithms were capable of retrieving the correct expression with a complete absence of irrelevant features. Overall, this task has proved to be a challenge for SR models and it shows that SR can possibly benefit from the application of a feature selection approach before fitting the data. To establish an expected achievable feature absence score and $R^{2}$ on these benchmarks, we applied off-the-shelf feature selection methods with gradient boosting as a comparison in Table III. In this case, the feature selection methods are set to select the correct number of variables (ten) which simplifies the feature selection problem. This table shows that SR alone is capable of selecting features better than simple feature selection methods. On the other hand, the use of feature importance combined with gradient boosting is on par with QLattice and pysr. The $R^{2}$ values, when using this specific method, are consistent with the top SR algorithms.

#### 3) Deceptive shortcuts:

**Fig. 6.**
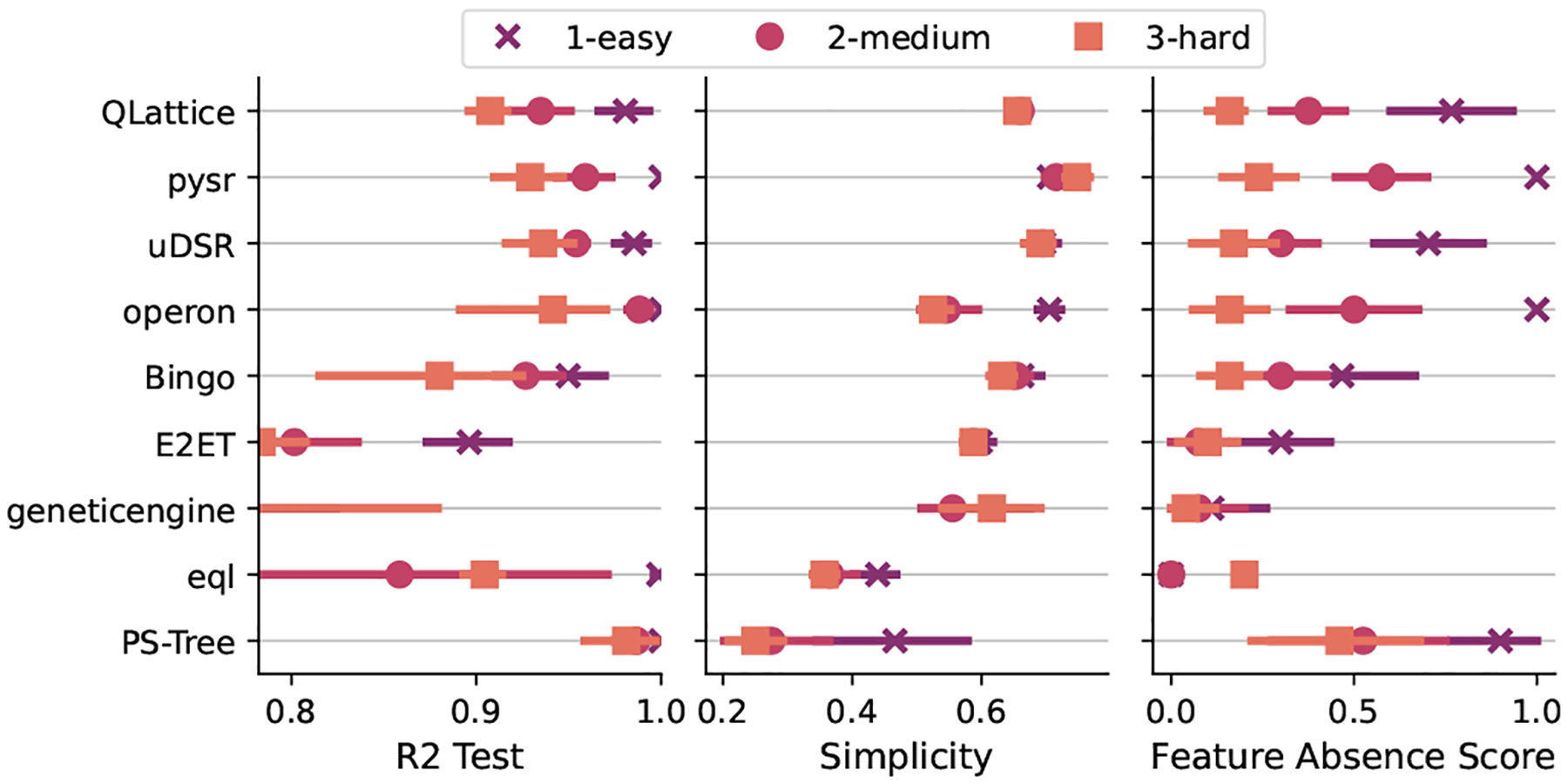
Distribution of ranks for the local optima task. Higher values are better.

For the local optima task (Fig. 6), we can see a similar behavior of the feature selection task w.r.t. the accuracy and simplicity scores. For the feature absence score, we observe a more spread distribution of the results, showing that there is a significant difference between the different difficulty levels. For the easy level, pySR and Operon used only relevant features in every run. One such example being the model:

$$f\left(x\right)=3.97x_{2}\left(0.19x_{1}+0.38x_{3}\right)+1.2x_{4}^{2},$$

that corresponds to the exact generating function. For the other difficulty levels, there were no perfect solutions. The best ones following the harmonic mean are:

$$f\left(x\right)=0.77x_{1}x_{2}+0.31x_{1}x_{4}x_{5}+1.52x_{2}x_{3}+1.2x_{4}^{2},$$

for the medium level with operon that also corresponds to the true model and

$$f\left(x\right)=x_{10}+1.53x_{2}x_{3}+1.23x_{4}^{2}+x_{6}+x_{9},$$

for the hard level with pySR that uses some of the local optima features to reach an almost perfect solution. As we can see from these results and Fig. 6, the noise plays an important role as it masquerades the importance of the true features. In other words, as much as pySR and Operon are competent to find the generating function, with too much noise in the target function, the noisy variables can be confused with the true variables. This task still presents a challenge and must be further investigate with more intermediate levels.

#### 4) Extrapolation accuracy:

**Fig. 7.**
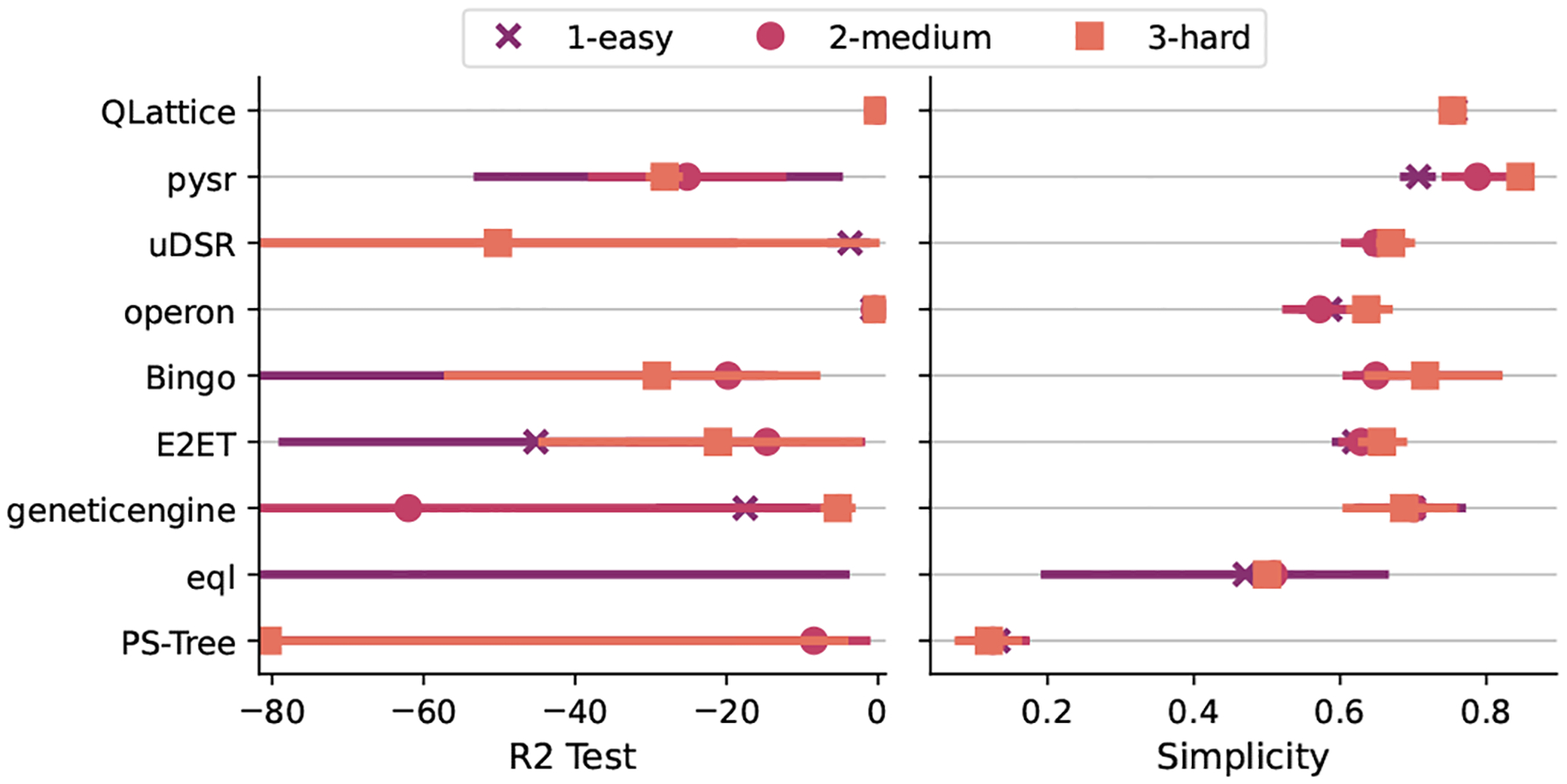
Distribution of ranks for the extrapolation task. Higher values are better.

The extrapolation task proved to be very challenging to SR algorithms, as we can see from Fig. 7. In this plot we can see that the obtained accuracy for most of the models were subpar, often with a negative $R^{2}$ for the test set, even for the easiest levels.

**Fig. 8.**
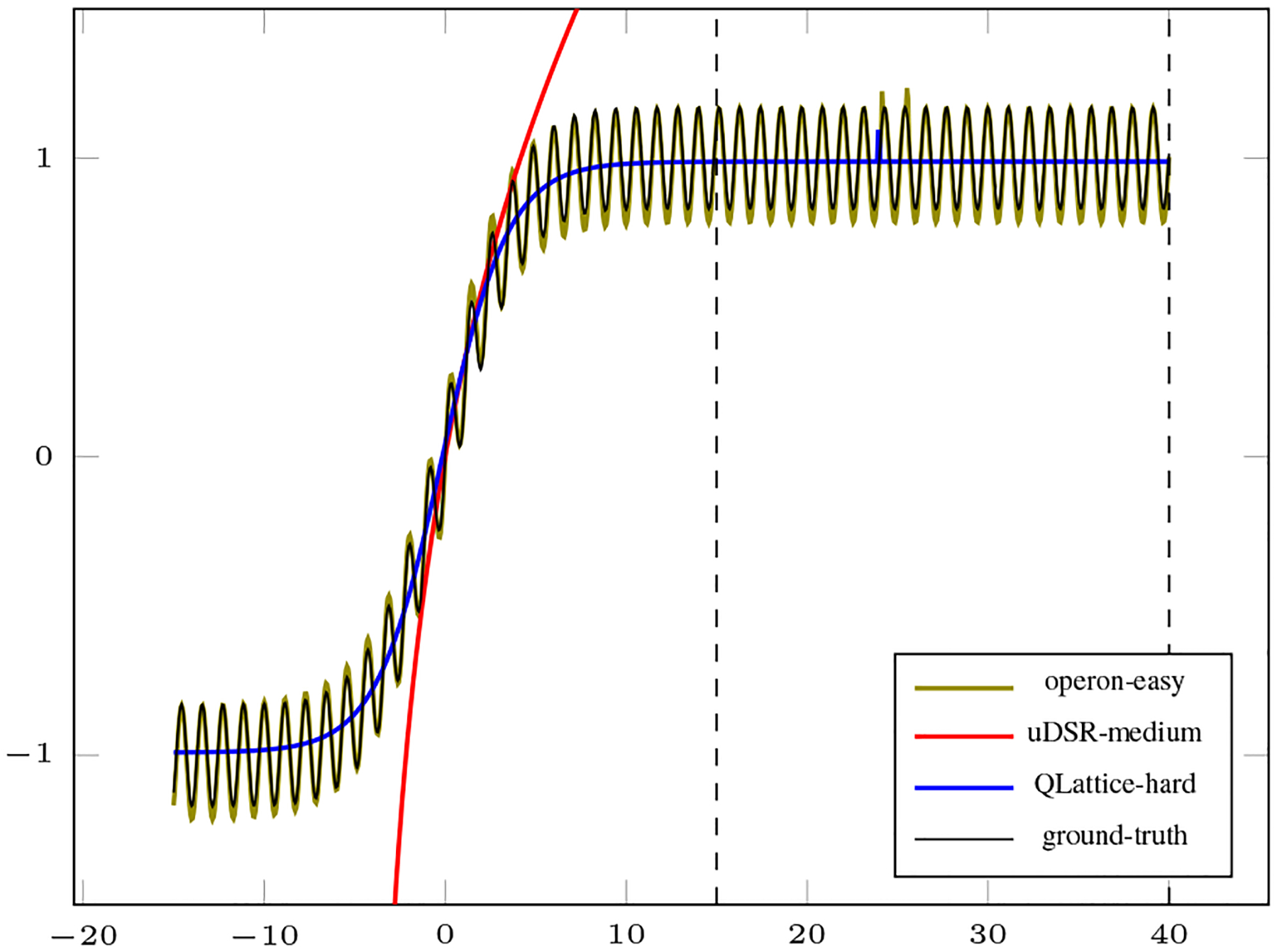
Selected solutions for the extrapolation task with different level of difficulties. Extrapolation region is between dashed lines.

To illustrate the behavior of models from different levels, we have selected the best harmonic mean of each difficulty level as illustrated in Fig. 8. We can see from this plot that Operon found a very close approximation to the original noiseless dataset, while QLattice returned models that approximated the error function without the sine term. The algorithm uDSR returned a function that approximated part of the interpolation region, but it was very different on the extrapolation data.

In summary, we can see that these algorithms were capable of capturing part of the target function missing one simple component. These results demand a broader investigation on why none of the algorithms were capable of including the sine function into the final expression.

#### 5) Sensitivity to noise:

**Fig. 9.**
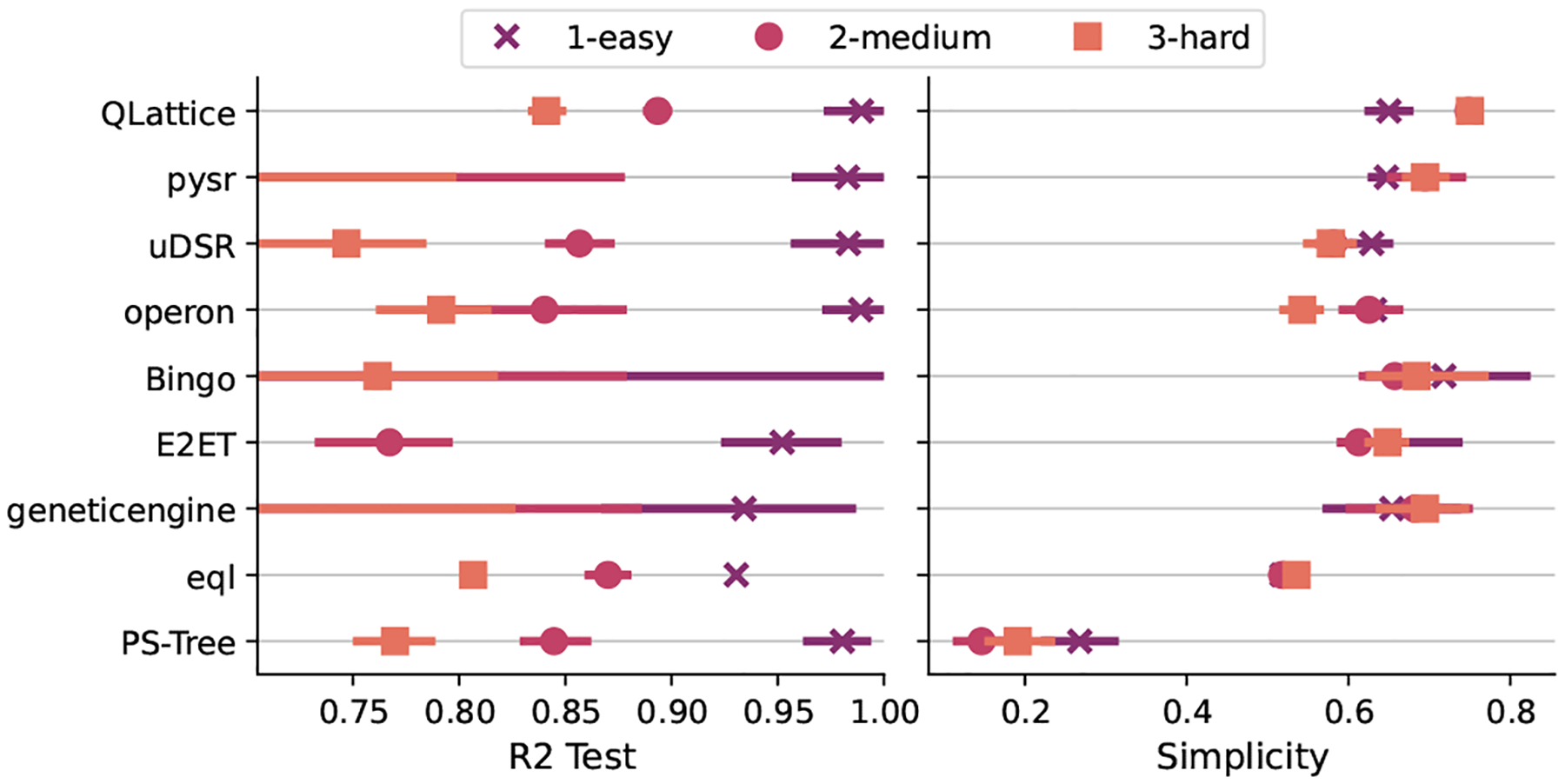
Distribution of ranks for the noise task. Higher values are better.

Finally, in Fig. 9 we can see the accuracy and simplicity scores for the sensitivity to noise task. From this plot we can clearly see the increase in difficulty as we increase the noise level. Even with the degradation of accuracy, almost every algorithm is capable of keeping the simplicity at a high score. Particularly for this task, QLattice, uDSR, operon, eql, and PS-Tree were capable of maintaining a relatively high accuracy across all difficulty levels.

### C. Real-world track

The expert was presented with a sequence of models with the necessary information to assess their evaluation. The evaluation screen contained information for the task (i.e., predicting cases, hospitalization, deaths), the event horizon, algorithm name, $R^{2}$ score, simplicity score, the regression model expression, a plot of the true time-series with the predicted time-series, and a plot of the predicted value versus the true value. The expert provided us with gestalt expert rating answering the question “I trust this model” with a scale from 1 (strongly disagree) to 5 (strongly agree). The final score is the harmonic mean of the model accuracy, simplicity score, and expert score.

**Fig. 10.**
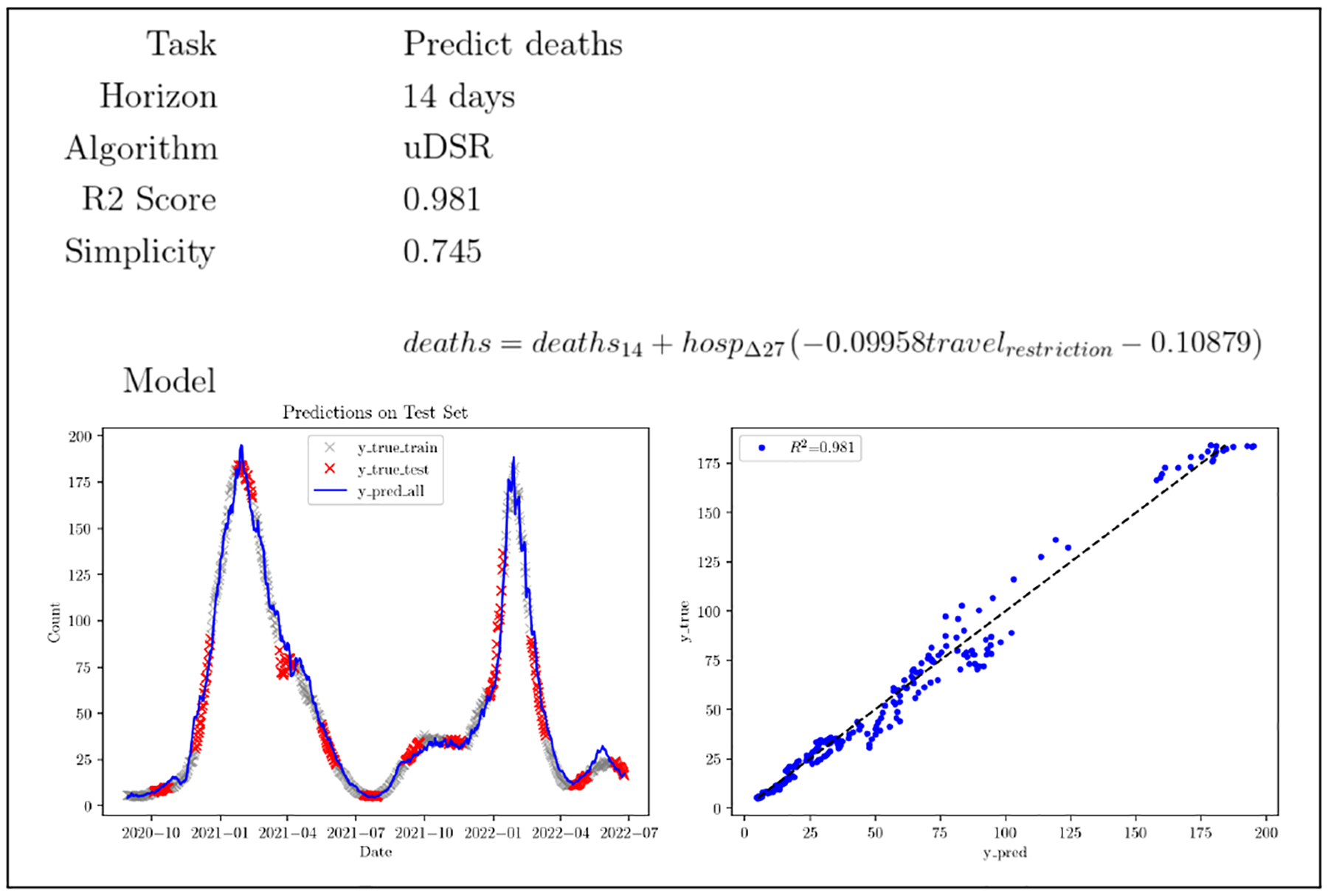
Sample presentation of a uDSR model used for expert assessment in the real-world track.

We report the results of this track in Table IV. In this track the best score was obtained by uDSR followed by QLattice and geneticengine. Fig. 10 shows a sample result for the prediction of the number of deaths using uDSR in the form presented to the expert for scoring. Although multiple approaches found good models for this task in terms of $R^{2}$ score, uDSR managed to return a comparably simple, plausible linear model with one interaction that generated good predictions.

## V. Discussion

Overall, this benchmark provided many insights about the current state of SR. In the remainder of this section, we focus on discussing different aspects of the benchmark itself. Throughout these benchmarks we noticed that there are still some gaps on the different degrees of difficulty for each task, filling these gaps is important to highlight the differences between algorithms and their limitations.

### A. Limitations of the benchmark

Regarding the limitations of this benchmark, one observation is that some algorithms performed better on one track and worse on another, or even they were better when observing one of the criteria and worse on another. Although we tried to balance this by using the harmonic mean, any aggregated final score is bound to obscure finer details of the results. Looking at the synthetic tracks, we can see that some algorithms are biased toward better accuracy, like Operon and QLattice, while others tend towards simpler models, like pysr. As some of these approaches employ different mechanisms to find a balance, would a better tweaking of the hyperparameters render a better result for those algorithms?

The simplicity measure only takes into account the size of the expression, but there are some constructs that hinders the interpretability of an expression without adding too much to the size. Consider the chain of nonlinear functions, like f(g(h(x)) that accounts for 4 nodes and a simpler expression like f(x)+h(x)*g(x), that accounts for 8 nodes. Even though the first one is smaller, the second one can be easier to reason about its behavior than the first. There are some examples of complexity measures such as in [33, 34].

Another issue is the computational budget, since we have a limited resource to run the experiments we have to limit the maximum runtime and repetitions. While the established runtime can be considered reasonable, a large number of repetitions could reduce some uncertainties about the results.

Finally, we should stress that there is hardly a single winner or a single approach that could be named SotA. We have to use an aggregation score to elect the winner; however, inspecting the results we can see that it is often the case that there are different winners on each track and each level.

### B. Overall difficulty of the tasks

Assessing the difficulty of the tasks can be challenging since it is not possible to test the problem instances before the benchmark due to the risk of biasing the results. As it turns out, some of the tasks proved to be really challenging. In this paper we controlled for the difficulty of the benchmarks by adding noise to the target values, introducing nonlinearity to the base function, inserting useless features, providing correlated and noisy features, and limiting the domain of the training data. Besides these approaches, there are some possibilities yet to be explored such as different data distributions, discontinuity, sparsity, and outliers.

For example, in the task of exact solution, just a few correct expressions were found and only on the easier and easy difficulties. This has two main reasons: i) any expression can be written using different alternative expressions such that the simplification procedure fails to match them; ii) the optimization of numerical parameters can lead to imprecision that causes the expression to be close but not equal to the ground-truth (as in Eq. 6).

The extrapolation task was also difficult for all of the approaches that approximated the error function well but without the sine term of the ground truth. On one hand, given that a certain dataset can have many different solutions inside the interpolation region that behaves differently outside this region (see [35]), we cannot expect the algorithm to make the right choice without any prior information. On the other hand, none of the algorithms could find a good solution even for the training data, revealing that the generating function was already challenging. Finally, the local optima and sensitivity to noise tasks provided a good balance between difficulties allowing us to verify that, while current SR algorithms can find a good solution with low noise data, the effect of the noise can have a large impact on the quality of the model. In the local optima task, we can see that the algorithms made a preference of maximizing simplicity even if that adds noise to the expression, this can be expected as it is an informed choice of choosing the expression with the smaller number of nodes even if that reduces the accuracy a bit.

Overall, it is important that we continuously adapt the benchmark problems using the information gained by past benchmarks to improve the difficulty range without introducing any bias towards any of the competitors. Additionally, a continuous adaptation would serve to avoid SR approaches evolving towards optimizing for these specific benchmarks, a common problem in many fields.

### C. Manual inspection is still a challenge

Challenges and opportunities remain for expert assessment of interpretability as conducted in the real-world track. First of all, the expert in the field had to evaluate many different models by looking at the analytic expression (whenever it had a reasonable size), the interpolation and extrapolation behavior of the time series, and the accuracy score. Comparing two or more models can already be challenging even for an expert in the field, as it requires them to first establish what is an interpretable model for them. Sometimes this ad-hoc evaluation may lead to some distortions on the result since there is no way to make a pairwise comparison between models without a quadratic increase the complexity of the evaluation task. Furthermore, expert assessments serve as “snapshot” evaluations of models in contrast to the other computable metrics that can be easily reproduced by researchers as SR methods evolve.

### D. Model Interpretability

Symbolic Regression is often referred to as a transparent modeling approach. While there is no consensus on how to measure interpretability, many measures and subjective evaluations have a role to play in gaining insight into processes through models. In this paper we used the size of the expressions (as a proxy) and the judgement of an expert (for the interpretability track) to verify whether SR returns interpretable expressions. In light of our results, we understand that this still requires a broader discussion within the SR community. For example, as argued in[36], a parametric regression model can be seen as a summarization of the available data in which the parameters have a very specific meaning in the context of the study. In this view, our desiderata is to generate a model with the fewest number of parameters necessary to achieve a good fit. This *compression efficiency* can be captured by techniques based on information theory such as minimium description length[37]. Even after achieving the desired properties, the interpretation still requires a domain expert to bring meaning to the results. In the context of XAI and Evolutionary Computation, SR plays just a partial role in the possibilities as argued in [38].

### E. Are we there yet?

In close, with a broad perspective of the results we can say, while we have witnessed many advances in this field, there are still some open challenges for the SR community.

One challenge is the support for a customised experience to the end-user, for example, specifying the degree of importance for a simpler solution when you have less accurate alternatives. In this same end, having alternative models that have a similar training accuracy could help the user to decide which one has the most probable extrapolation behavior. Additionally, as much as an analytic solution can be considered more interpretable than an opaque model, the SR tool should provide supporting information in the form of uncertainties quantification, visual behavior, and additional data such as partial derivatives and properties of the model. A minor issue is the runtime of these algorithms, as they mostly rely on evolutionary algorithms, they are usually slower than common regression algorithms.

Specifically on the assessed tasks, there are still room for improvement on the treatment of noise and feature selection. While most algorithms were capable of adequately handling noise and to remove some of the not useful features, the noise data still had an impact on the accuracy reducing the $R^{2}$ by up to 20%. Also, the removal of not needed features can lead to even simpler models that are more informative to the end-user.

## VI. Conclusion

This paper proposed an extension to SRBench benchmark for Symbolic Regression, called SRBench++ and provided an in-depth analysis of initial results obtained for 12 SotA SR algorithms. The main purpose of the benchmark was to understand how modern SR algorithms handle common challenges in data science for regression analysis and to have an indication of where the current SotA stands. For this purpose we have created different tasks divided in separate tracks: i) rediscovery of exact expressions; ii) feature selection; iii) avoiding noisy local optima; iv) accuracy in extrapolation; v) noisy data. All these tasks are relevant not only to regression analysis but also for the interpretability of the models. We also held an additional track with the evaluation of the generated models on real-world data by an expert in their respective field.

We note that, overall, that there is no dominating algorithm that returns the best model in every criteria within the benchmark. These results help to understand the advantage and disadvantages of each approach and it can move the field forward to better algorithms. As this benchmark was evaluated in the form of a competition, each participant chose how to perform the hyperparameter setting (if done at all) and this choice may also have affected the final results for each algorithm. Also, the choice of programming language may affect the computational budget available as faster implementations have the chance to evaluate more solutions. These two aspects are out of the scope of a general benchmark, but SRBench++ is open sourced, any author can run experiments with different implementations and settings (see [39]). In the near future, we plan to release new editions of this benchmark with new and improved tasks from additional domains and areas of interest. We will also use different evaluation criteria that can help us to better understand how each algorithm stands with respect to distinct challenges.

## Supplementary Material

Supplement

## Figures and Tables

**Table I:** Generating functions for the rediscovery of exact expression task and their corresponding degree of difficulty.

Function	Difficulty	Generating Function
$f_{1}(x)$	Easier	$0.4x_{1}x_{2}-1.5x_{1}+2.5x_{2}+1$
$f_{2}(x)$	Easy	$f_{1}(x)+\text{log}\left(30x_{3}^{2}\right)$
$f_{3}(x)$	Medium	$\frac{f_{1}(x)}{0.2\left(x_{1}^{2}+x_{2}^{2}\right)+1}$
$f_{4}(x)$	Hard	$\frac{f_{1}(x)+5.5\;\text{sin}\;\left(x_{1}+x_{2}\right)}{0.2\left(x_{1}^{2}+x_{2}^{2}\right)+1}$

**Table II:** Summary of the methods submitted to the benchmark. For method class, we use: EA=Evolutionary algorithm (e.g., GP), DL=Deep learning, Mix=Combination of multiple classes.

Name	Class	Code	Brief description
Bingo [20]	EA	URL	Evolves general acyclic graphs with a linear representation. Includes coefficient fine-tuning, algebraic simplification, and coevolution of fitness predictors.
E2ET [8]	DL	URL	A pre-trained transformer that predicts SR models directly from the data. Predicted models are then fine-tuned and the best is returned.
EQL [21]	DL	URL	Consists of a fully-differentiable, shallow neural network that contains SR operators as activations. The $L^{0}$ loss is used to prune the network.
GeneticEngine [22]	EA	URL	Uses strongly-typed and grammar-guided GP. For this benchmark, no domain-specific information was needed for the grammar.
NSGA-DCGP	EA	URL	Combines differentiable cartesian GP [23] with the non-dominated sorting genetic algorithm II (NSGA-II) to simultaneously discover short and accurate models.
Operon [24]	EA	URL	C++-coded GP, where fine-tuning is realized with the Levenberg-Marquardt algorithm. It is paired with Optuna for tuning search hyper-parameters.
PS-Tree [25]	MIX	URL	Combines decision trees, GP, and ridge regression within the evolutionary process. It is capable of tackling the SR problem in a piece-wise fashion.
PySR [26]	EA	URL	Uses tree-based expressions, tournament selection, and local leaf search. It further uses multiple populations during the search.
QLattice [27]	EA	URL	Uses a probability distribution, updated over the iterations, to sample increasingly better solutions. It includes fine-tuning and more.
TaylorGP [28]	EA	URL	Combines GP with Taylor polynomial approximations. It uses Taylor expansions to identify polynomial features and decompose the problem.
uDSR [29]	MIX	URL	Unified framework for SR that combines: recursive problem simplification, neural-guided search, large-scale pre-training, sparse linear regression, and GP.
GP_ZGD_ [30]	EA	URL	Koza-style canonical GP with the addition of stochastic gradient descent to tune coefficients during the evolution.

**Table III:** Baseline for the feature selection task using different feature selection algorithms. $R^{2}$ is calculated on the test using grad. boosting with the selected variables. Feat. importance selects the features based on the weights associated by gradient boost, rfe dose the same but recursively picking one by one, kbest chooses the top-k based on statistical tests, variance picks those with the most variance.

Difficulty	Selector	Feature Absence Score	$R^{2}$
easy	Feature importance	0.90	0.64
	kbest	0.50	−0.14
	rfe	0.50	0.66
	variance	0.00	0.63
medium	Feature importance	0.90	0.62
	kbest	0.50	−0.14
	rfe	0.50	0.63
	variance	0.00	0.63
hard	Feature importance	0.90	0.57
	kbest	0.50	−0.14
	rfe	0.50	0.60
	variance	0.00	0.57

**Table IV:** Results for the real-world track.

Rank	Algorithm	Score	Rank	Algorithm	Score
1	uDSR	5.75	5	Bingo	4.66
2	QLattice	5.21	6	pySR	4.17
3	geneticengine	4.99	7	PS-Tree	3.15
4	Operon	4.80	8	E2ET	2.72

## Footnotes

1
https://cavalab.org/srbench

2
https://github.com/cavalab/srbench/tree/Competition2022

3
https://cavalab.org/srbench/competition-2022/

## References

[1] Koza JR, Genetic Programming: On the Means of Programming Computers by Means of Natural Selection. MIT Press, 1992.

[2] Cranmer M, Sanchez Gonzalez A, Battaglia P, Xu R, Cranmer K, Spergel D, and Ho S, “Discovering symbolic models from deep learning with inductive biases,” Advances in Neural Information Processing Systems, vol. 33, pp. 17 429–17 442, 2020.

[3] Hernandez A, Balasubramanian A, Yuan F, Mason SAM, and Mueller T, “Fast, accurate, and transferable many-body interatomic potentials by symbolic regression,” npj Computational Materials, vol. 5, no. 1, p. 112, Nov 2019.

[4] La Cava WG, Lee PC, Ajmal I, Ding X, Solanki P, Cohen JB, Moore JH, and Herman DS, “A flexible symbolic regression method for constructing interpretable clinical prediction models,” npj Digital Medicine, vol. 6, no. 1, pp. 1–14, Jun. 2023. 37277550 10.1038/s41746-023-00833-8 PMC10241925

[5] Virgolin M and Pissis SP, “Symbolic regression is NP-hard,” Transactions on Machine Learning Research, 2022. [Online]. Available: https://openreview.net/forum?id=LTiaPxqe2e

[6] Petersen BK, Larma ML, Mundhenk TN, Santiago CP, Kim SK, and Kim JT, “Deep symbolic regression: Recovering mathematical expressions from data via risk-seeking policy gradients,” arXiv preprint arXiv:1912.04871, 2019.

[7] Biggio L, Bendinelli T, Neitz A, Lucchi A, and Parascandolo G, “Neural symbolic regression that scales,” in International Conference on Machine Learning. PMLR, 2021, pp. 936–945.

[8] Kamienny P-A, d’Ascoli S, Lample G, and Charton F, “End-to-end symbolic regression with transformers,” arXiv preprint arXiv:2204.10532, 2022.

[9] La Cava W, Orzechowski P, Burlacu B, de França FO, Virgolin M, Jin Y, Kommenda M, and Moore JH, “Contemporary symbolic regression methods and their relative performance,” arXiv preprint arXiv:2107.14351, 2021. PMC11074949 38715933

[10] Orzechowski P, La Cava W, and Moore JH, “Where are we now? A large benchmark study of recent symbolic regression methods,” in Proceedings of the 2018 Genetic and Evolutionary Computation Conference, ser. GECCO’18, Apr. 2018.

[11] La Cava W, Singh TR, Taggart J, Suri S, and Moore JH, “Learning concise representations for regression by evolving networks of trees,” in International Conference on Learning Representations, ser. ICLR, 2019.

[12] Shwartz-Ziv R and Armon A, “Tabular data: Deep learning is not all you need,” Information Fusion, vol. 81, pp. 84–90, 2022.

[13] Friedman JH, “Greedy function approximation: a gradient boosting machine,” Annals of statistics, pp. 1189–1232, 2001.

[14] Bomarito GF, Leser PE, Strauss N, Garbrecht K, and Hochhalter J, “Automated learning of interpretable models with quantified uncertainty,” arXiv preprint arXiv:2205.01626, 2022.

[15] Rudin C, “Stop explaining black box machine learning models for high stakes decisions and use interpretable models instead,” Nature Machine Intelligence, vol. 1, no. 5, pp. 206–215, 2019. 10.1038/s42256-019-0048-x PMC9122117 35603010

[16] Roland W, Marschik C, Kommenda M, Haghofer A, Dorl S, and Winkler S, “Predicting the non-linear conveying behavior in single-screw extrusion: A comparison of various data-based modeling approaches used with cfd simulations,” International Polymer Processing, vol. 36, no. 5, pp. 529–544, 2021. [Online]. Available: 10.1515/ipp-2020-4094

[17] Romano JD, Le TT, La Cava W, Gregg JT, Goldberg DJ, Chakraborty P, Ray NL, Himmelstein D, Fu W, and Moore JH, “PMLB v1.0: An open-source dataset collection for benchmarking machine learning methods,” Bioinformatics, 2022. 10.1093/bioinformatics/btab727 PMC8756190 34677586

[18] Olson RS, La Cava W, Orzechowski P, Urbanowicz RJ, and Moore JH, “PMLB: A Large Benchmark Suite for Machine Learning Evaluation and Comparison,” BioData Mining, 2017. 10.1186/s13040-017-0154-4 PMC5725843 29238404

[19] Meurer A, Smith CP, Paprocki M, Čertík O, Kirpichev SB, Rocklin M, Kumar A, Ivanov S, Moore JK, Singh S , “Sympy: symbolic computing in python,” PeerJ Computer Science, vol. 3, p. e103, 2017.

[20] Randall DL, Townsend TS, Hochhalter JD, and Bomarito GF, “Bingo: a customizable framework for symbolic regression with genetic programming,” in Proceedings of the Genetic and Evolutionary Computation Conference Companion, 2022, pp. 2282–2288.

[21] Sahoo S, Lampert C, and Martius G, “Learning equations for extrapolation and control,” in International Conference on Machine Learning. PMLR, 2018, pp. 4442–4450.

[22] Espada G, Ingelse L, Canelas P, Barbosa P, and Fonseca A, “Data types as a more ergonomic frontend for grammar-guided genetic programming,” in Proceedings of the 21st ACM SIGPLAN International Conference on Generative Programming: Concepts and Experiences, GPCE 2022, Auckland, New Zealand, December 6–7, 2022, Scholz B and Kameyama Y, Eds. ACM, 2022, pp. 86–94. [Online]. Available: 10.1145/3564719.3568697

[23] Izzo D, Biscani F, and Mereta A, “Differentiable genetic programming,” in Genetic Programming: 20th European Conference, EuroGP 2017, Amsterdam, The Netherlands, April 19–21, 2017, Proceedings 20. Springer, 2017, pp. 35–51.

[24] Burlacu B, Kronberger G, and Kommenda M, “Operon C++ an efficient genetic programming framework for symbolic regression,” in Proceedings of the 2020 Genetic and Evolutionary Computation Conference Companion, 2020, pp. 1562–1570.

[25] Zhang H, Zhou A, Qian H, and Zhang H, “Ps-tree: A piecewise symbolic regression tree,” Swarm and Evolutionary Computation, vol. 71, p. 101061, 2022.

[26] Cranmer M, “Pysr: Fast & parallelized symbolic regression in python/julia,” 2020.

[27] Broløs KR, Machado MV, Cave C, Kasak J, Stentoft-Hansen V, Batanero VG, Jelen T, and Wilstrup C, “An approach to symbolic regression using feyn,” arXiv preprint arXiv:2104.05417, 2021.

[28] He B, Lu Q, Yang Q, Luo J, and Wang Z, “Taylor genetic programming for symbolic regression,” arXiv preprint arXiv:2205.09751, 2022.

[29] Landajuela M, Lee C, Yang J, Glatt R, Santiago CP, Aravena I, Mundhenk TN, Mulcahy G, and Petersen BK, “A unified framework for deep symbolic regression,” in Advances in Neural Information Processing Systems, Oh AH, Agarwal A, Belgrave D, and Cho K, Eds., 2022. [Online]. Available: https://openreview.net/forum?id=2FNnBhwJsHK

[30] Dick G, Owen CA, and Whigham PA, “Feature standardisation and coefficient optimisation for effective symbolic regression,” in Proceedings of the 2020 Genetic and Evolutionary Computation Conference, ser. GECCO ‘20. New York, NY, USA: Association for Computing Machinery, 2020, p. 306–314. [Online]. Available: 10.1145/3377930.3390237

[31] Kronberger G, “Local optimization often is ill-conditioned in genetic programming for symbolic regression,” in 2022 24th International Symposium on Symbolic and Numeric Algorithms for Scientific Computing (SYNASC). IEEE, 2022, pp. 304–310.

[32] de Franca FO and Kronberger G, “Reducing overparameterization of symbolic regression models with equality saturation,” in Proceedings of the Genetic and Evolutionary Computation Conference, 2023, pp. 1064–1072.

[33] Kommenda M, Beham A, Affenzeller M, and Kronberger G, “Complexity measures for multi-objective symbolic regression,” in International Conference on Computer Aided Systems Theory. Springer, 2015, pp. 409–416.

[34] Virgolin M, De Lorenzo A, Medvet E, and Randone F, “Learning a formula of interpretability to learn interpretable formulas,” in International Conference on Parallel Problem Solving from Nature. Springer, 2020, pp. 79–93.

[35] De Franca FO, “Fighting underspecification in symbolic regression with fitness sharing,” in Proceedings of the Companion Conference on Genetic and Evolutionary Computation, 2023, pp. 551–554.

[36] Russeil E, de França FO, Malanchev K, Burlacu B, Ishida EE, Leroux M, Michelin C, Moinard G, and Gangler E, “Multi-view symbolic regression,” arXiv preprint arXiv:2402.04298, 2024.

[37] Bartlett DJ, Desmond H, and Ferreira PG, “Exhaustive symbolic regression,” IEEE Transactions on Evolutionary Computation, 2023.

[38] Bacardit J, Brownlee AE, Cagnoni S, Iacca G, McCall J, and Walker D, “The intersection of evolutionary computation and explainable ai,” in Proceedings of the Genetic and Evolutionary Computation conference companion, 2022, pp. 1757–1762.

[39] Burlacu B, “Gecco’2022 symbolic regression competition: Post-analysis of the operon framework,” in Proceedings of the Companion Conference on Genetic and Evolutionary Computation, 2023, pp. 2412–2419.

