# Supplementary material for: SRBench++ : principled benchmarking of symbolic regression with domain-expert interpretation
Source: IEEE Trans Evol Comput. Author manuscript; Available in PMC 2025 Aug 4. (PMC12321164; doi:10.1109/tevc.2024.3423681)
Supplement: Supplement [file NIHMS2053059-supplement-Supplement.pdf]

Supplementary Material for SRBench++: principled benchmarking of symbolic regression with domain-expert interpretation

F. O. de Franca*, M. Virgolin†, M. Kommenda¶, M. S. Majumder‡, M. Cranmer‖, G. Espada††, L. Ingelse††, A. Fonseca††, M. Landajuela‡‡, B. Petersen‡‡, R. Glatt‡‡, N. Mundhenk‡‡, C. S. Lee‡‡, J. D. Hochhalter[x], D. L. Randall[x], P. Kamienny[xi], H. Zhang[xii], G. Dick[xiii], A. Simon[xiv], B. Burlacu¶, Jaan Kasak[xv], Meera Machado[xv], Casper Wilstrup[xv], W. G. La Cava‡

I. Participation instructions

In order to participate, the candidates had to provide an installation script that could be run without administrative permissions on a Linux system inside a *conda* [1] environment [1], a *metadata.yml* document describing their submission, and a Python script called *regression.py* containing a *scikit-learn* [2] Regressor object [2], and a function returning a *sympy*[3] [3] compatible string of the SR model. For each run, a candidate SR algorithm had a pre-specified time budget of 1 hour for datasets up to 1000 samples and 10 hours for datasets up to 10000 samples. Candidates were responsible for ensuring that the runtime of their algorithm (including choices of hyperparameter optimization) would not exceed the budget. In order to make comparisons as fair as possible, participants were not given advance notice of what datasets would be used for the competition. They knew only that datasets would follow the format in the Penn Machine Learning Benchmark (PMLB) [4, 5], and that datasets from PMLB would be used in the qualifying stage.

*Center for Mathematics, Computation and Cognition (CMCC), Heuristics, Analysis and Learning Laboratory (HAL), Federal University of ABC, Santo Andre, Brazil.
†Evolutionary Intelligence group, Centrum Wiskunde & Informatica, Science Park 123, Amsterdam, Netherlands.
‡Computational Health Informatics Program, Boston Children's Hospital, Harvard Medical School, Boston, USA.
¶Heuristic and Evolutionary Algorithms Laboratory (HEAL), University of Applied Sciences Upper Austria, Hagenberg, Austria.
‖Center for Computational Astrophysics, Flatiron Institute and Department of Astrophysical Sciences of Princeton University, USA.
††LASIGE, Faculdade de Ciências, Universidade de Lisboa, Lisboa, Portugal.
‡‡Computational Engineering Division, Lawrence Livermore National Laboratory, Livermore, USA.
[x]University of Utah, Department of Mechanical Engineering, Utah, USA.
[xi]Meta, FAIR, France.
[xii]Victoria University of Wellington, School of Engineering and Computer Science, New Zealand.
[xiii]University of otago, Department of Information Science, New Zealand.
[xiv]Institut für Angewandte Physik, Universität Tübingen; Max Planck Institute for Intelligent Systems, Tübingen, Germany
[xv]Abzu AI, Orient Plads 1, Nordhavn 2150, Denmark
Preprint Under Review. Corresponding author: W. G. La Cava (email: william.lacava@childrens.harvard.edu)

[1]https://www.anaconda.com/
[2]https://scikit-learn.org/
[3]https://www.sympy.org/en/index.html

TABLE I
Datasets used during the qualification stage.

dataset	# samples	# variables
197_cpu_act	8192	22
215_2dplanes	40768	11
227_cpu_small	8192	13
556_analcatdata_apnea2	475	4
557_analcatdata_apnea1	475	4
564_fried	40768	11
573_cpu_act	8192	22
218_house_8L	22784	9
225_puma8NH	8192	9
294_satellite_image	6435	37
666_rmftsa_ladata	508	11
579_fri_c0_250_5	250	6
586_fri_c3_1000_25	1000	26
590_fri_c0_1000_50	1000	51
593_fri_c1_1000_10	1000	11
595_fri_c0_1000_10	1000	11
654_fri_c0_500_10	500	11
581_fri_c3_500_25	500	26
582_fri_c1_500_25	500	26
584_fri_c4_500_25	500	26

With the objective of making the competition accessible and reproducible by external peers, we created a new branch in the SRBench repository[4] with instructions on how to add a new method and how to run the competition on a local machine. Following these requirements, it is possible for the general public to reproduce the competition and experiment with these SR algorithms by cloning the competition branch and following the installation and execution instructions.

II. Datasets of the qualification stage

Table I shows the selected datasets used in the qualification stage. They were chosen as a subset of the PMLB datasets that the top-10 algorithms from srbench performed equally well.

III. Ranking

As we used multiple evaluation criteria for each track: R^2, simplicity, and a task-specific score for each tasks, we computed the aggregated rank as the harmonic means of the ranks for each criterion for n different data-sets.

[4]https://github.com/cavalab/srbench/tree/Competition2022

$$\text{agg\_rank} = \frac{n}{\sum_{i=1}^{n} \frac{1}{\text{rank}_i}} \quad (1)$$

The harmonic mean imposes that, to be highly ranked, you cannot have a low rank in any of the criteria. This avoids the situation that an SR algorithm returns a very simple model with low R^2 and still ranks high among the competitors.

The simplicity score is defined as $\text{round}(-\log_5(s), 1)$, where s is the number of nodes in the expression tree after being simplified by *sympy* [3], and $\text{round}(x, n)$ rounds the value x to the n-th place. Rounding was introduced to provide some tolerance for similarly-sized expressions. Fig. 1 demonstrates how this simplicity score relates to the number of nodes in a tree.

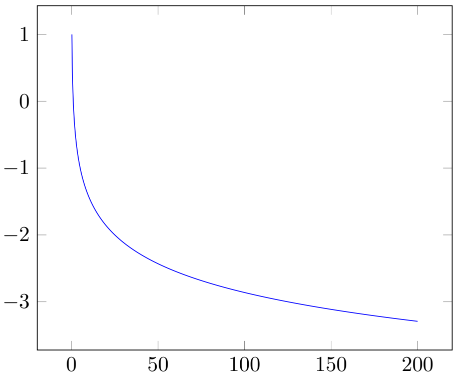

Fig. 1. Plot of the simplicity score.

IV. Description of the methods

Bingo [6] implements a GP strategy for SR with a few distinctions from typical generic-programming-based SR. First, expressions are represented as directed acyclic graphs, rather than trees. Fitness calculations of the expressions involves a two step procedure: the directed acyclic graphs are algebraically simplified , and a gradient-based local optimization is performed on numeric constants. Lastly, coevolution of fitness predictors [7] is used for large datasets ($>$ 1200 datapoints). The several hyperparameters of the method were tuned prior to submission (details in [6]) to place emphasis on obtaining low-complexity models.

E2ET [8] involves utilizing a generative model to generate analytical expressions based on given observations. Specifically, a transformer-based neural network that is conditioned on the observations, generates a distribution over expressions. The process of sampling from this distribution involves utilizing auto-regressive decoding of expression symbols, which includes variables, operator tokens, and tokenized floats. To further enhance the generated expressions, the constants are optimized using the BFGS algorithm. This search process is highly efficient and takes only approximately one second for a single forward-pass.

PS-Tree [9], or Piecewise SR Tree, is a piecewise regression method that combines decision trees, GP, and ridge regression to divide the feature space to multiple subspaces and create a set of nonlinear models in subspaces for making prediction. The proposed PS-Tree aims to achieve high-predictive performance while retaining a reasonable level of interpretability. Specifically, PS-Tree evolves a set of GP trees under a multi-objective optimization framework to evolve a common set of non-linear features for all ridge regression models in each subspaces simultaneously. Then, it dynamically constructs a decision tree during the evolution process to automatically determine the optimal partition scheme based on the runtime information. By doing so, PS-Tree is able to achieve high accuracy for solving piecewise regression tasks with reasonable interpretability.

QLattice [10] is a framework for SR that mixes GP and numerical optimization. It is accessible through the Python library feyn. The QLattice manages a pool of models, and samples new ones via edits on the best performing models in the pool. Model performance is only evaluated after optimizing the free parameters in each model by an internally-tuned gradient descent algorithm on the training data. The available edit operations on models are chosen among by means of weighted sampling, where the weights have been optimized by Abzu. Training data with categorical features is also inherently supported, where each possible category is assigned a weight arrived at through fitting the training data. In this competition, the best model as selected by the Bayesian Information Criterion was returned from the final set of models.

TaylorGP [11] combines SR with Taylor polynomial approximations. The method uses Taylor expansions both to identify polynomial features and to decompose problems into simpler tasks.

EQL [12] is a fully differentiable neural network for SR, which permits it to be used in contexts requiring differentiable end-to-end computations. It works by approximating the equation by a shallow network with non-linear activation functions, which can be adapted to the task at hand. While sparsity can be achieved through different means, for this competition we optimized the stochastic L^0 loss of the expansion parameters and selected the best expression based on the quality of the fit and the simplicity of the expression according to Pareto optimality. Once the expression is fixed, the appearing constants are finally optimized via BFGS.

GeneticEngine [13] is a Python framework that supports different flavours of Strongly-Typed and Grammar-Guided GP. The entry used a tree-based representation, as it produces higher quality crossovers than array based approaches . Grammars are defined using standard Python classes and inheritance, allowing domain-specific knowledge to be easily incorporated into the search process. While this is the main advantage of the approach, in this competition no domain-specific information was used in the grammar.

Operon [14] is a C++ framework for SR with a focus on performance and scalability. It supports single- and multi-objective tree-based GP and local tuning of model coefficients via the Levenberg-Marquardt algorithm. Parallelism is implemented at the level of individual recombination events using a flexible graph-based task model [15]. Additionally, data-level parallelism is employed during tree evaluation. The Python module, PyOperon, makes this functionality available as a

scikit-learn estimator. For this competition, Operon is paired with Optuna [16] for hyperparameter tuning the best model from the last-generation Pareto front is returned.

PySR [17] and its accompanying backend, SymbolicRegression.jl, is an evolutionary optimization algorithm for tree-based expressions based on tournament selection and local leaf searches. PySR emphasizes performance and is very configurable—it does not place a particular prior over expression space, or define any standard operators or functional forms. PySR achieves efficient evaluation by JIT-compiling operators into fused SIMD kernels, and by multi-threading at the population-level. The full Pareto front of expressions is recorded during the search, but for this competition, the equation with the greatest improvement in accuracy along the Pareto front is returned.

uDSR [18], or Unified Deep SR, is a modular, unified framework for SR that combines five solution strategies: recursive problem simplification [19], neural-guided search [20], large-scale pre-training for problem generalization [21], sparse linear regression using nonlinear basis functions [22], and combination of evolutionary search with learning [23]. By viewing these modules as connected but non-overlapping components within an algorithmic framework, uDSR is able to harness their unique strengths and address their respective limitations. The modular setup even works for a subset of the proposed modules and allows for the easy exchange of individual modules with new methods should they become available. uDSR is implemented using the open-source Deep Symbolic Optimization framework [5].

GP_{ZGD} [24] is a straightforward 'out-of-the-box' implementation of Koza-style canonical GP [25] for SR, with the addition of stochastic gradient descent (SGD) applied to model coefficients during evolution. To make the SGD more effective, Z-score standardisation is applied to all regressors. Hyperparameter tuning over Koza's canonical settings was performed to optimise performance to SR as outlined in [26]. GP_{ZGD} was a late submission to the competition, so it was only assessed in the qualification stage of the competition.

NSGA-DCGP [27] is a method combining differentiable cartesian GP [27] with NSGA-II with the objectives of mimizing error and complexity.

V. Supplemental Results

A. Wall-clock running time

Regarding the runtime, in Fig. 2 we can see that half of the participants used the full budget to find the best solution. As most of them are population-based search algorithms, it is a sane strategy to maximize the number of evaluations in the hopes of finding a good local optima. EQL, E2ET and PS-Tree adopts a different strategy to search for the expression so that there is no mechanism to stop premature convergence. As such, once it reaches a local optimum, the search stops.

B. Statistical Tests

Synthetic track post-hoc statistical test results are given in Fig. 3.

[5]https://github.com/brendenpetersen/deep-symbolic-optimization

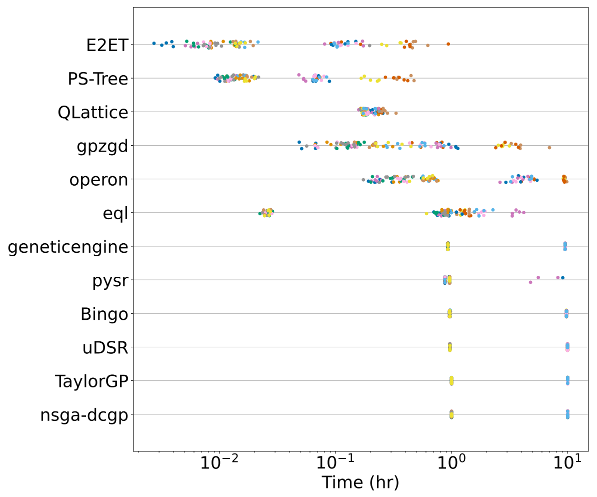

Fig. 2. Runtime of each SR method for every tested dataset during the qualification stage. Each colored dot is a dataset. Note that methods were limited to one hour of total time for datasets with less than 1000 samples and limited to ten hours for larger datasets.

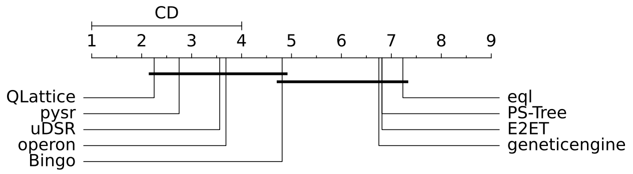

Fig. 3. Critical difference diagram for algorithm results in Stage 1. Statistical comparisons are based on the average of the harmonic mean of the problem criteria using Nemenyi test with $\alpha = 0.05$ as a post-hoc test.

C. Additional expert rating details

To conduct expert rating in a controlled manner, a slide deck was prepared containing one slide for each model generated by the algorithms for predicting cases, hospitalizations, and death. The rater was first provided a summary of the COVID task setup, training and test set development, and model evaluation. They were then instructed as follows: "Please rate your trust in these models on a scale of 1 to 5, with a higher rating indicating stronger trust." The subsequent slides contained model equations, metrics, and visualizations of performance as shown in the main text.

During expert evaluation of trust scores, the rater had the option to leave additional notes alongside their numerical ratings. The decision of whether to leave comments was left up to the expert. For the task of 14 day look-ahead prediction of case counts, and a model developed by `PS-Tree` received the following additional comment: "Predictions are best for this algorithm, but black box inherently makes it less trustworthy despite performance". In this instance the model complexity was too large to display on the page.

Following the initial evaluation, the expert rater provided additional narrative explanations of their ratings for each model of death counts. The presented slides, expert ratings, and narratives are show in Figs. 4 to 11. In addition to individual narratives, we compile the model performance, simplicity score, and expert rating for predictions of cases, hospitalizations, and

deaths in Table II. In general, we observe that expert ratings of trust depended foremost on model predictive performance, assessed both visually and numerically, and secondarily on the ability to intepret the returned model form. Characteristics of interpretability that appear frequently include assessments of the direction of effect of individual terms, the modularity of model components, and the intelligibility of interactions between variables.

VI. Data Availability

The full results files of the competition are available from the following url: https://zenodo.org/record/6842176. The code to run the analysis is available from https://cavalab.org/srbench/competition-guide/.

References

[1] Anaconda, "Anaconda software distribution." [Online]. Available: https://anaconda.com/

[2] F. Pedregosa, G. Varoquaux, A. Gramfort, V. Michel, B. Thirion, O. Grisel, M. Blondel, P. Prettenhofer, R. Weiss, V. Dubourg, J. VanderPlas, A. Passos, D. Cournapeau, M. Brucher, M. Perrot, and E. Duchesnay, "Scikit-learn: Machine learning in python," *J. Mach. Learn. Res.*, vol. 12, pp. 2825–2830, 2011. [Online]. Available: https://dl.acm.org/doi/10.5555/1953048.2078195

[3] A. Meurer, C. P. Smith, M. Paprocki, O. Čertík, S. B. Kirpichev, M. Rocklin, A. Kumar, S. Ivanov, J. K. Moore, S. Singh *et al.*, "Sympy: symbolic computing in python," *PeerJ Computer Science*, vol. 3, p. e103, 2017.

[4] J. D. Romano, T. T. Le, W. La Cava, J. T. Gregg, D. J. Goldberg, P. Chakraborty, N. L. Ray, D. Himmelstein, W. Fu, and J. H. Moore, "PMLB v1.0: An open-source dataset collection for benchmarking machine learning methods," *Bioinformatics*, 2022.

[5] R. S. Olson, W. La Cava, P. Orzechowski, R. J. Urbanowicz, and J. H. Moore, "PMLB: A Large Benchmark Suite for Machine Learning Evaluation and Comparison," *BioData Mining*, 2017.

[6] D. L. Randall, T. S. Townsend, J. D. Hochhalter, and G. F. Bomarito, "Bingo: a customizable framework for symbolic regression with genetic programming," in *Proceedings of the Genetic and Evolutionary Computation Conference Companion*, 2022, pp. 2282–2288.

[7] M. D. Schmidt and H. Lipson, "Coevolution of fitness predictors," *IEEE Transactions on Evolutionary Computation*, vol. 12, no. 6, pp. 736–749, 2008.

[8] P.-A. Kamienny, S. d'Ascoli, G. Lample, and F. Charton, "End-to-end symbolic regression with transformers," *arXiv preprint arXiv:2204.10532*, 2022.

[9] H. Zhang, A. Zhou, H. Qian, and H. Zhang, "Ps-tree: A piecewise symbolic regression tree," *Swarm and Evolutionary Computation*, vol. 71, p. 101061, 2022.

[10] K. R. Broløs, M. V. Machado, C. Cave, J. Kasak, V. Stentoft-Hansen, V. G. Batanero, T. Jelen, and C. Wilstrup, "An approach to symbolic regression using feyn," *arXiv preprint arXiv:2104.05417*, 2021.

[11] B. He, Q. Lu, Q. Yang, J. Luo, and Z. Wang, "Taylor genetic programming for symbolic regression," *arXiv preprint arXiv:2205.09751*, 2022.

[12] S. Sahoo, C. Lampert, and G. Martius, "Learning equations for extrapolation and control," in *International Conference on Machine Learning*. PMLR, 2018, pp. 4442–4450.

[13] G. Espada, L. Ingelse, P. Canelas, P. Barbosa, and A. Fonseca, "Data types as a more ergonomic frontend for grammar-guided genetic programming," in *Proceedings of the 21st ACM SIGPLAN International Conference on Generative Programming: Concepts and Experiences, GPCE 2022, Auckland, New Zealand, December 6-7, 2022*, B. Scholz and Y. Kameyama, Eds. ACM, 2022, pp. 86–94. [Online]. Available: https://doi.org/10.1145/3564719.3568697

[14] B. Burlacu, G. Kronberger, and M. Kommenda, "Operon C++ an efficient genetic programming framework for symbolic regression," in *Proceedings of the 2020 Genetic and Evolutionary Computation Conference Companion*, 2020, pp. 1562–1570.

[15] T.-W. Huang, D.-L. Lin, C.-X. Lin, and Y. Lin, "Taskflow: A lightweight parallel and heterogeneous task graph computing system," *IEEE Transactions on Parallel and Distributed Systems*, vol. 33, no. 6, pp. 1303–1320, 2022.

[16] T. Akiba, S. Sano, T. Yanase, T. Ohta, and M. Koyama, "Optuna: A next-generation hyperparameter optimization framework," in *Proceedings of the 25th ACM SIGKDD International Conference on Knowledge Discovery and Data Mining*, 2019.

[17] M. Cranmer, "Pysr: Fast & parallelized symbolic regression in python/julia," 2020.

[18] M. Landajuela, C. Lee, J. Yang, R. Glatt, C. P. Santiago, I. Aravena, T. N. Mundhenk, G. Mulcahy, and B. K. Petersen, "A unified framework for deep symbolic regression," in *Advances in Neural Information Processing Systems*, A. H. Oh, A. Agarwal, D. Belgrave, and K. Cho, Eds., 2022. [Online]. Available: https://openreview.net/forum?id=2FNnBhwJsHK

[19] S.-M. Udrescu, A. Tan, J. Feng, O. Neto, T. Wu, and M. Tegmark, "Ai feynman 2.0: Pareto-optimal symbolic regression exploiting graph modularity," *Advances in Neural Information Processing Systems*, vol. 33, pp. 4860–4871, 2020.

[20] B. K. Petersen, M. L. Larma, T. N. Mundhenk, C. P. Santiago, S. K. Kim, and J. T. Kim, "Deep symbolic regression: Recovering mathematical expressions from data via risk-seeking policy gradients," *arXiv preprint arXiv:1912.04871*, 2019.

[21] L. Biggio, T. Bendinelli, A. Neitz, A. Lucchi, and G. Parascandolo, "Neural symbolic regression that scales," in *International Conference on Machine Learning*. PMLR, 2021, pp. 936–945.

[22] S. L. Brunton, J. L. Proctor, and J. N. Kutz, "Discovering governing equations from data by sparse identification of nonlinear dynamical systems," *Proceedings of the national academy of sciences*, vol. 113, no. 15, pp. 3932–3937, 2016.

TABLE II
MODEL EVALUATIONS FOR THE REAL-WORLD TRACK.

Algorithm	Cases R2	Cases Simplicity	Cases Expert	Hospitalizations R2	Hospitalizations Simplicity	Hospitalizations Expert	Deaths R2	Deaths Simplicity	Deaths Expert
Bingo	0.70	0.65	3	0.79	0.62	4	0.98	0.64	4
E2ET	0.21	0.51	2	0.77	0.58	2	0.92	0.53	2
PS-Tree	0.97	0.00	3	0.94	0.04	3	0.99	0.02	3
Qlattice	0.87	0.56	4	0.86	0.58	5	0.99	0.56	5
geneticengine	0.64	0.71	3	0.8	0.75	5	0.98	0.71	4
operon	0.70	0.51	5	0.84	0.71	4	0.98	0.56	4
pysr	0.72	0.75	3	0.76	0.75	4	0.97	0.69	3
uDSR	0.75	0.58	4	0.82	0.62	5	0.98	0.75	4

Task	Predict deaths
Horizon	14 days
Algorithm	Bingo
R2 Score	0.979
Simplicity	0.623
Model	$deaths = 1.012 deaths_{14} - (0.19844 deaths_{14} hosp_{\Delta 24} - 0.19844 deaths_{18} + 39.688 deaths_{\Delta 22} + 1.62721 hosp_{\Delta 16} - 33.7348) / \sqrt{cases_{26}}$

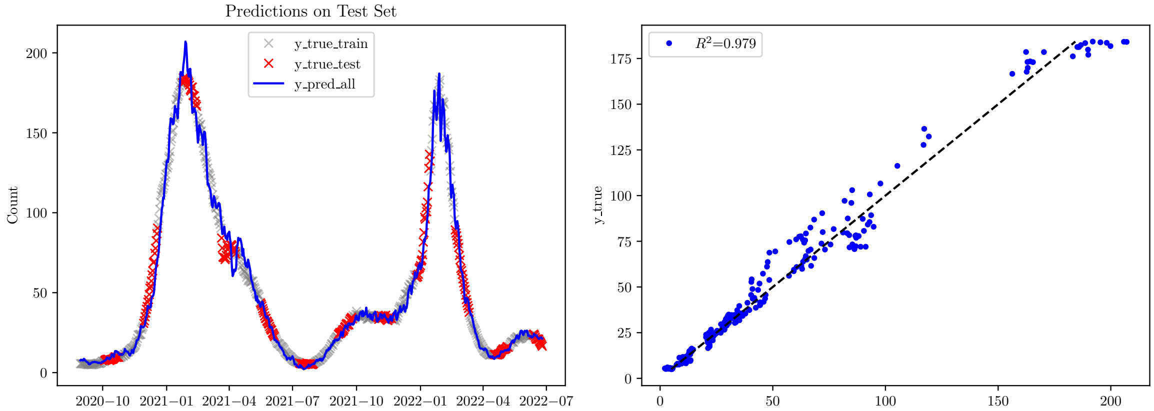

Trust Rating: 4
Assessment: Over-prediction of first peak in deaths near February 2021, but good R2 and simplicity otherwise. Linear terms are easy to interpret but effect directions and interaction terms (14-day deaths and change in 24 day hospitalizations, 26 day case counts) are unclear.

Fig. 4. Expert rating of a Bingo model predicting death counts for the real-world track.

[23] T. Mundhenk, M. Landajuela, R. Glatt, C. P. Santiago, B. K. Petersen *et al.*, "Symbolic regression via deep reinforcement learning enhanced genetic programming seeding," *Advances in Neural Information Processing Systems*, vol. 34, pp. 24 912–24 923, 2021.

[24] G. Dick, C. A. Owen, and P. A. Whigham, "Feature standardisation and coefficient optimisation for effective symbolic regression," in *Proceedings of the 2020 Genetic and Evolutionary Computation Conference*, ser. GECCO '20. New York, NY, USA: Association for Computing Machinery, 2020, p. 306–314. [Online]. Available: https://doi.org/10.1145/3377930.3390237

[25] J. R. Koza, *Genetic Programming: On the Means of Programming Computers by Means of Natural Selection*. MIT Press, 1992.

[26] G. Dick, "Genetic programming, standardisation, and stochastic gradient descent revisited: Initial findings on srbench," in *Proceedings of the Genetic and Evolutionary Computation Conference Companion*, ser. GECCO '22. New York, NY, USA: Association for Computing Machinery, 2022, p. 2265–2273. [Online]. Available: https://doi.org/10.1145/3520304.3534040

[27] D. Izzo, F. Biscani, and A. Mereta, "Differentiable genetic programming," in *Genetic Programming: 20th European Conference, EuroGP 2017, Amsterdam, The Netherlands, April 19-21, 2017, Proceedings 20*. Springer, 2017, pp. 35–51.

Task	Predict deaths
Horizon	14 days
Algorithm	E2ET
R2 Score	0.919
Simplicity	0.585
Model	$deaths = -3.0 \cdot 10^{-5} hosp_{18} + 0.16196 hosp_{23} - 9.67 \sin\left(0.00081 hosp_{19} + 0.00283 hosp_{20} + 0.26896 hosp_{22} - 0.05983 hosp_{23} + 11.76734 + \frac{10.96797}{0.02 hosp_{15} - 9.95}\right) - 11.08318$

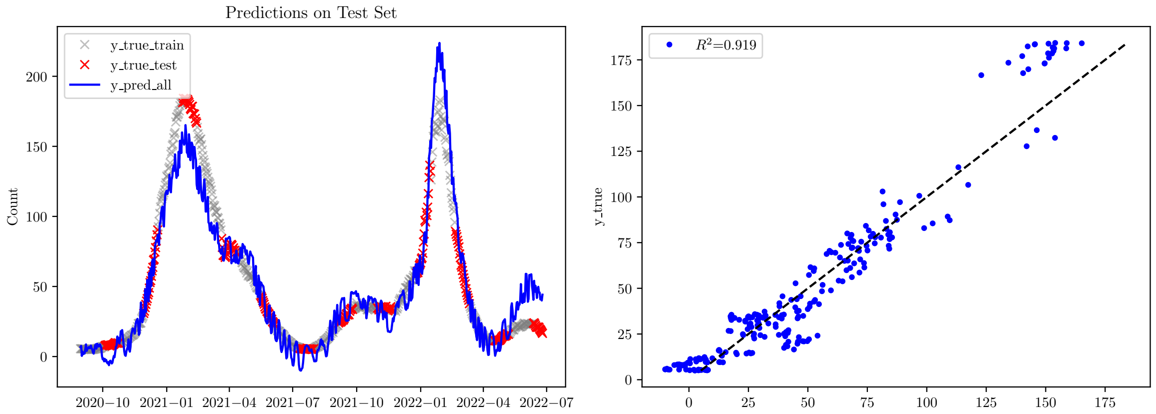

Trust Rating: 2
Assessment: Second worst R^2. Over and under-estimation of peak deaths. Model form is large and overly complex, including very small parameter values.

Fig. 5. Expert rating of an E2ET model predicting death counts for the real-world track.

Task	Predict deaths
Horizon	14 days
Algorithm	PS-Tree
R2 Score	0.994
Simplicity	0.0
Model	black-box

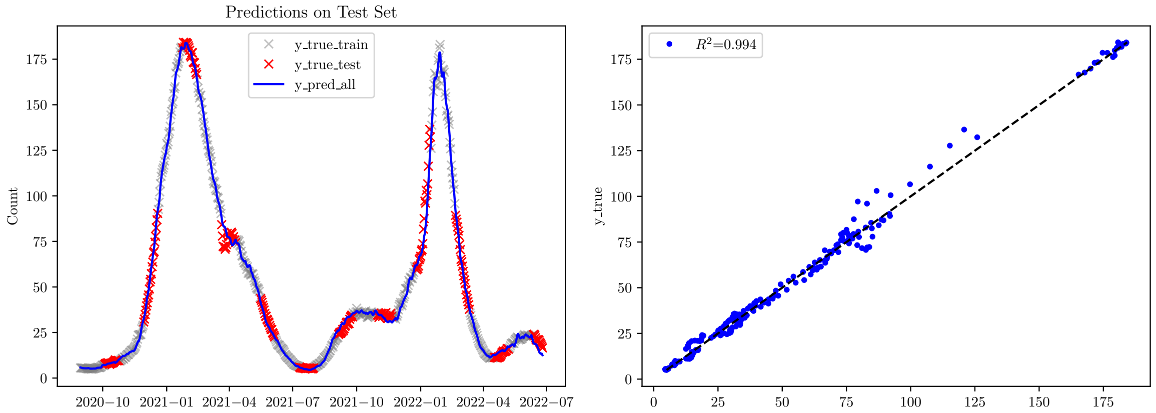

Trust Rating: 3
Assessment: Model shows good performance, but is uninterpretable. Because it is not interpretable at all, we can't say whether it will perform well under other conditions.

Fig. 6. Expert rating of a PS-Tree model predicting death counts for the real-world track.

Task	Predict deaths
Horizon	14 days
Algorithm	QLattice
R2 Score	0.993
Simplicity	0.564
Model	$deaths = 328.411 - 326.833 e^{-0.004(-0.025 deaths_{27} - 1)^2 - 2.0 \tanh^2(0.00056 hosp_{14} + 0.01758)}$

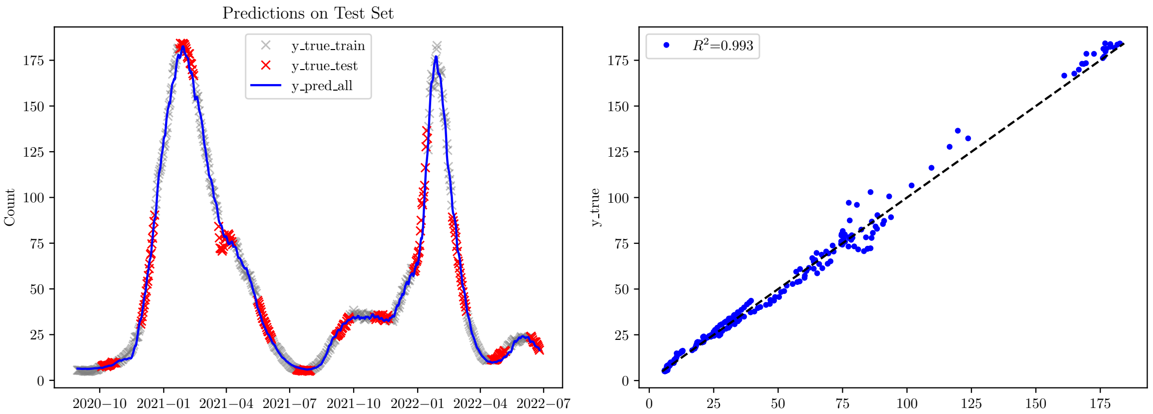

Trust Rating: 5

Assessment: Excellent R^2 capturing peak case counts and decent simplicity with a somewhat dense model form. Model predicts monotonic increases in deaths with increases in two week hospitalizations and 27 day deaths. Hospitalizations are observed to lag reported deaths by approximately this amount. Squared hyperbolic tangent on hospitalizations count is assumed to scale and saturate this feature since hospitalization counts are often an order of magnitude higher than deaths. Interpretability is limited by the complex functions/operators but is considered an improvement over the complete black-box results.

Fig. 7. Expert rating of a QLattice model predicting death counts for the real-world track.

Task	Predict deaths
Horizon	14 days
Algorithm	geneticengine
R2 Score	0.975
Simplicity	0.679
Model	$deaths = (deaths_{14} + 0.1 deaths_{tot})\,(deaths_{14} + deaths_{21} mask_{mandate} + hosp_{14})\,/ deaths_{tot}$

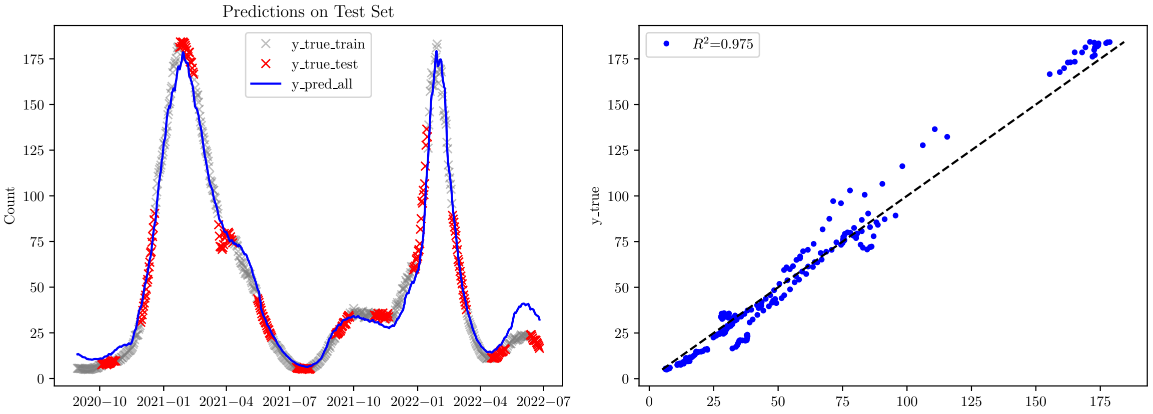

Trust Rating: 4

Assessment: Good R^2 and simplicity overall but over-prediction of second peak deaths count. Model predicts future death counts from previous reports, and also includes a death rate feature (division by total deaths). Interaction between 21 day deaths and mask mandate is unclear.

Fig. 8. Expert rating of a geneticengine model predicting death counts for the real-world track.

Task	Predict deaths
Horizon	14 days
Algorithm	operon
R2 Score	0.985
Simplicity	0.547
Model	$deaths = -\frac{0.022 cases_{15}}{\sqrt{deaths_{22}^2 + 0.111}} + \frac{0.009 cases_{18}}{\sqrt{deaths_{14}^2 + 0.189}} + 1.565 deaths_{14} - 0.587 deaths_{22} + 0.41 deaths_{\Delta 17} - 0.196 hosp_{\Delta 21} + \frac{0.004 hosp_{\Delta 21}}{\sqrt{CompleteVaccinationsDaily^2 travel_{restriction}^2}} - 0.065 hosp_{\Delta 27} + 2.652$

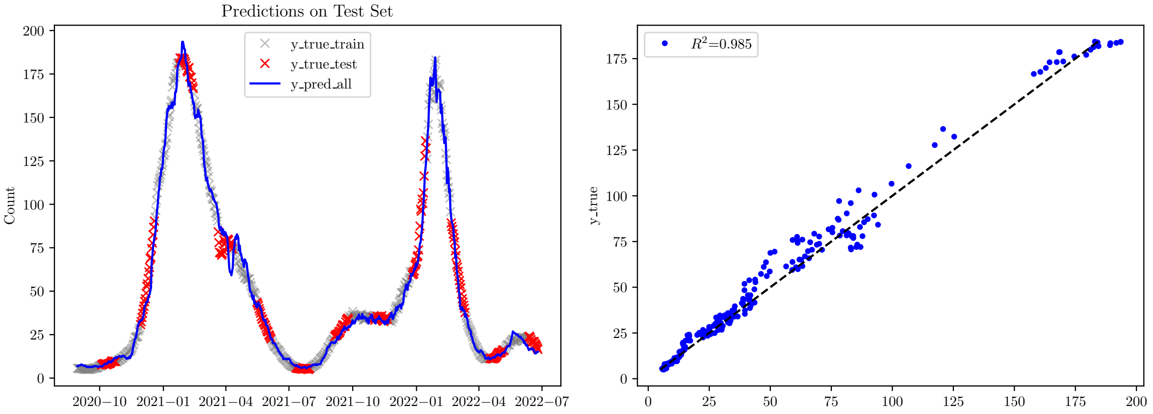

Trust Rating: 4

Assessment: Good R^2 but under-prediction of latter half of final peak death counts. Model performs well across values of the outcome and some linear terms are interpretable. Fraction terms limit overall interpretability, including the directions of effect for simpler terms.

Fig. 9. Expert rating of an operon model predicting death counts for the real-world track.

Task	Predict deaths
Horizon	14 days
Algorithm	pysr
R2 Score	0.975
Simplicity	0.679
Model	$deaths = deaths_{14}\left(\frac{hosp_{14}}{0.417deaths_{14}+0.417hosp_{20}} - 1.032\right)$

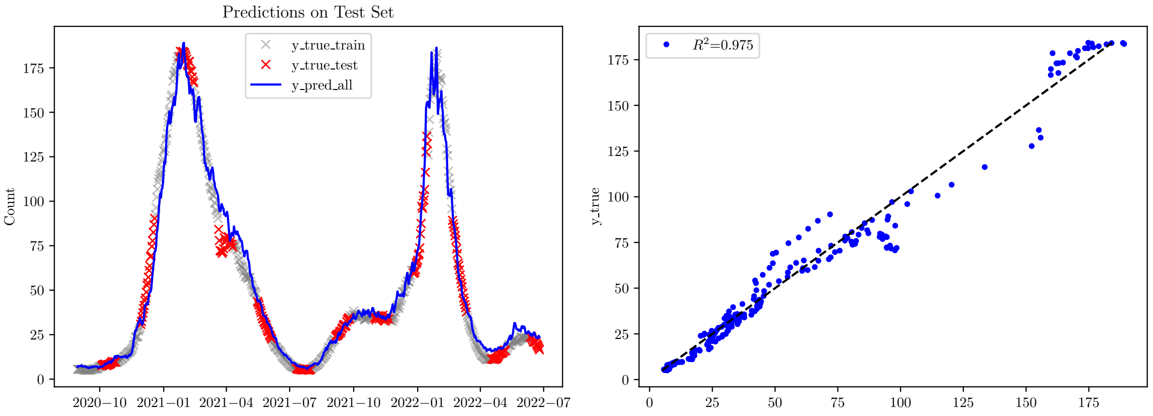

Trust Rating: 3
Assessment: Slightly lower R2 performance compared to other models. Noticeable divergence of predictions for similar death counts (right plot) lowers rating.

Fig. 10. Expert rating of a pysr model predicting death counts for the real-world track.

Task	Predict deaths
Horizon	14 days
Algorithm	uDSR
R2 Score	0.981
Simplicity	0.745
Model	$deaths = deaths_{14} + hosp_{\Delta 27}\left(-0.1travel_{restriction} - 0.109\right)$

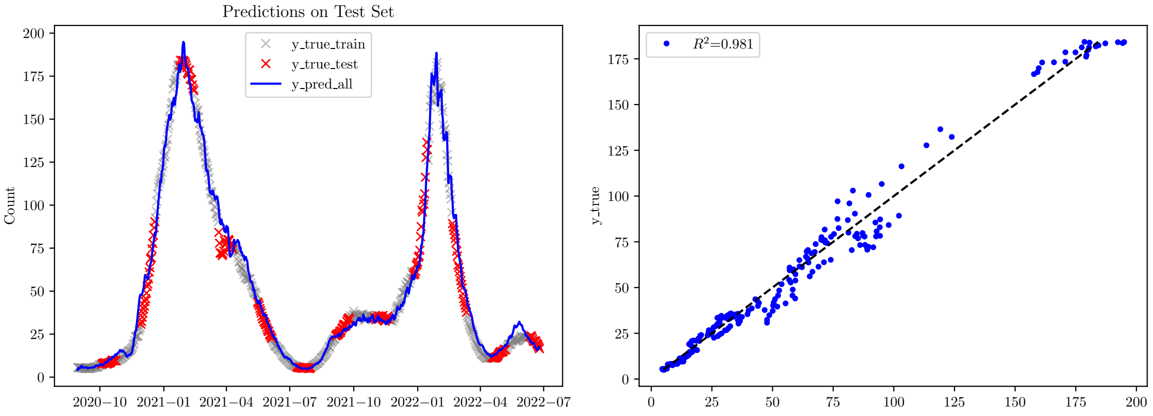

Trust Rating: 4
Assessment: Simplest model for deaths with good performance. On the last peak of death counts, it over predicts; this is probably what is driving down R^2. Little bit of overestimation on the first peak, but not pronounced given that it seems to be only for a short duration. Effect directions for 14 day deaths (increase), change in 27 day hospitalizations (increase), and current travel restriction (decrease) appear correct. Interaction between travel restriction and hospitalization count changes is interesting, since travel restrictions may impact patient flow, wait times, admission/discharge decisions.

Fig. 11. Expert rating of a uDSR model predicting death counts for the real-world track.
